# A Threshold-Limited Fluorescence Probe for Viscosity

**DOI:** 10.3389/fchem.2019.00342

**Published:** 2019-05-14

**Authors:** Zuhai Lei, Kai Xin, Shaobing Qiu, Liling Hou, Xiangming Meng, Youjun Yang

**Affiliations:** ^1^State Key Laboratory of Bioreactor Engineering, Shanghai Key Laboratory of Chemical Biology, School of Pharmacy, East China University of Science and Technology, Shanghai, China; ^2^Shanghai Key Laboratory of Molecular Catalysis and Innovative Materials, State Key Laboratory of Molecular Engineering of Polymers and iChem, Department of Chemistry, Fudan University, Shanghai, China; ^3^Department of Chemistry, Anhui University, Hefei, China

**Keywords:** threshold-limited, probe, viscosity, two-photon, fluorescence

## Abstract

Viscosity of body fluid is an established biomarker of pathological conditions. Abnormality of cellular viscosity occurs when cells are challenged with external stresses. Small molecule probes to assess the viscosity are sought after for both disease diagnostics and basic studies. Fluorescence based probes are particular attractive due to their potentials for convenient and high spatiotemporal resolution microscopic monitoring of biological samples. The dyes with a floppy push-pull backbone or dyes with a rotatable substituent exhibits a viscosity responsive fluorescence enhancement and therefore viable viscosity probes. The scaffold of the existing viscosity probes contains typically one such floppy site. Therefore, they typically linearly respond to log(viscosity). We argue that minor viscosity fluctuation could potentially be physiological as the biological system is dynamic. We wish to develop a type of conceptually-new, threshold-limited viscosity probes, to complement the existing probes. Such probes do not exhibit a fluorescence enhancement when challenged with minor and presumably physiological enhancement of viscosity. When the viscosity is higher than a certain threshold, their fluorescence turns on. We hypothesize that a dye with two far-apart floppy sites could potentially yield such a threshold-limited signal and designed **VPZ2** and **VPZ3**. Through spectral titration, **VPZ3** was found to yield the desired threshold-limited signal. **VPZ3** was suitable for *in vitro* bioimaging of viscosity under one-photon or two-photon excitation. **VPZ3** is potentially useful in many downstream applications. Future work includes fine-tune of the threshold to allow tailored limit for fluorescence turn-on to better meet the need of different applications. Besides the implications in the real-world applications, the design concept could also be translated to design of alternative substrates.

## Introduction

Viscosity is a biophysical parameter of homeostasis (Tsien, 1989; Balkwill et al., 2012; Wang et al., 2017). By altering the molecular diffusion kinetics, biomolecular trafficking, lipid fluidity, and protein conformational rate, all physiological processes including enzymatic activity, energy metabolism, and signal transduction are affected (Miyamoto et al., 1990; Uribe and Smpedro, 2003; Boric et al., 2012; Liu et al., 2014; Sekhar et al., 2014). Abnormal fluctuation of microenvironmental viscosity is found to be reliable biomarker of underlying diseases or stresses (Aydemir et al., 2008; Harisa, 2014; Kasperczyk et al., 2014; Herranz et al., 2018). Oxidative burst can alter the membrane fluidity through lipid peroxidation and hence disrupts its function (Richter, 1987; Hormel et al., 2013). Lysosome storage disorders are associated with increased local viscosity (Platt et al., 2012; Devany et al., 2018). Viscosity is also vital to maintain the mitochondrial network organization and energy metabolism (Mecocci et al., 1997). Hyperviscosity, or macroscopic high blood viscosity, is found with patients of many blood diseases, such as myeloma, leukemia, anemia, and sepsis (Gustine et al., 2016). Therefore, fluorescent probes for viscosity are in need for both basic biomedical studies and disease diagnosis to monitor the viscosity of complex biological systems (Haidekker and Theodorakis, 2007; Kuimova et al., 2008; Sutharsan et al., 2010; Kuimova, 2012; Wang et al., 2013; López-Duarte et al., 2014; Yang et al., 2014; Chen et al., 2015; Vyšniauskas et al., 2015, 2016; Lee et al., 2016; Ren et al., 2016; Su et al., 2016; Zhu et al., 2016; Klymchenko, 2017; Lyubov et al., 2017; Ning et al., 2017;Song et al., 2017).

**Figure 1:**
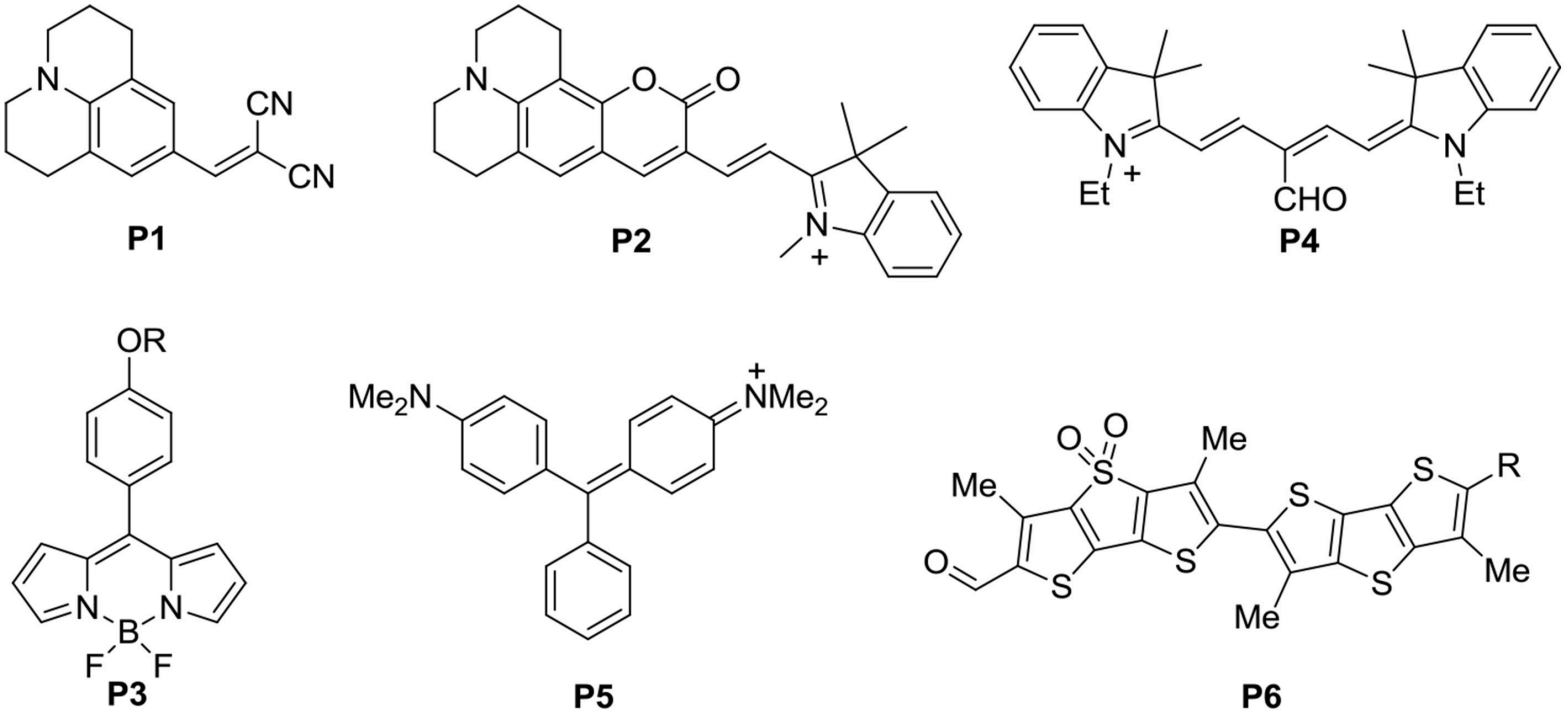
Structures of a number of classic molecular rotors (**1**–**6**).

The capability of a dye to sense environmental viscosity originates from its excited state dynamics, including non-radiative rotational deactivation and radiative deactivation (Klymchenko, 2017). For a fluorophore exhibiting a high degree of rotational freedom, it is typically non-fluorescent. When the rotational freedom is restricted, possibility of radiative deactivation enhances. The structural freedom of the fluorophore may come from rotatable bonds of the push-pull backbone, as seen in **P1** (Loutfy and Arnold, 1982), and **P2** (Cui et al., 2019) (Figure 1). Or, a group may be installed to a rigid push-pull backbone via a single bond to construct a molecular rotor, e.g., **P3** (Kuimova et al., 2008). Or, it could be a flexible push-pull backbone installed with a rotatable group, e.g., **P4** (Peng et al., 2011), **P5** (Babendure et al., 2003), and **P6** (Colom et al., 2018) (Figure 1). Typically, these molecular rotors respond linearly to log(viscosity). Minor enhancement of microscopic viscosity could be physiological considering the dynamic nature of a biological system. Therefore, we are interested in development of a new class of molecular rotors which does not respond to minor enhancement of viscosity, until the viscosity surpasses a certain threshold limit. Such threshold-limited molecular rotors could potentially be very useful in disease diagnostics. The existence of one site of high rotational freedom in the scaffold of a fluorophore is required to yield a viscosity-sensitive fluorescence enhancement. We propose that two such sites are warranted to exhibit threshold-limited response to viscosity. Intuitively, minor enhancement of viscosity may restrict the rotational freedom of one site, leaving the other site unaffected to quench the fluorescence of the fluorophore. When the viscosity is higher than a certain limit, the chances of simultaneous restriction of both sites becomes possible and fluorescence enhancement should be noticeable. Also, sterics should be present in the scaffold of such a probe to minimize unintended fluorescence turn-on by aggregation or unselective binding with native biomacromolecules (Lei et al., 2017).

Herein, we report the synthesis, spectral titrations and proof-of-concept bioimaging of threshold-limited fluorescent probe (**VPZ1-3**) for microscopic viscosity.

## Results and Discussions

### Compound Synthesis

**Scheme 1:**
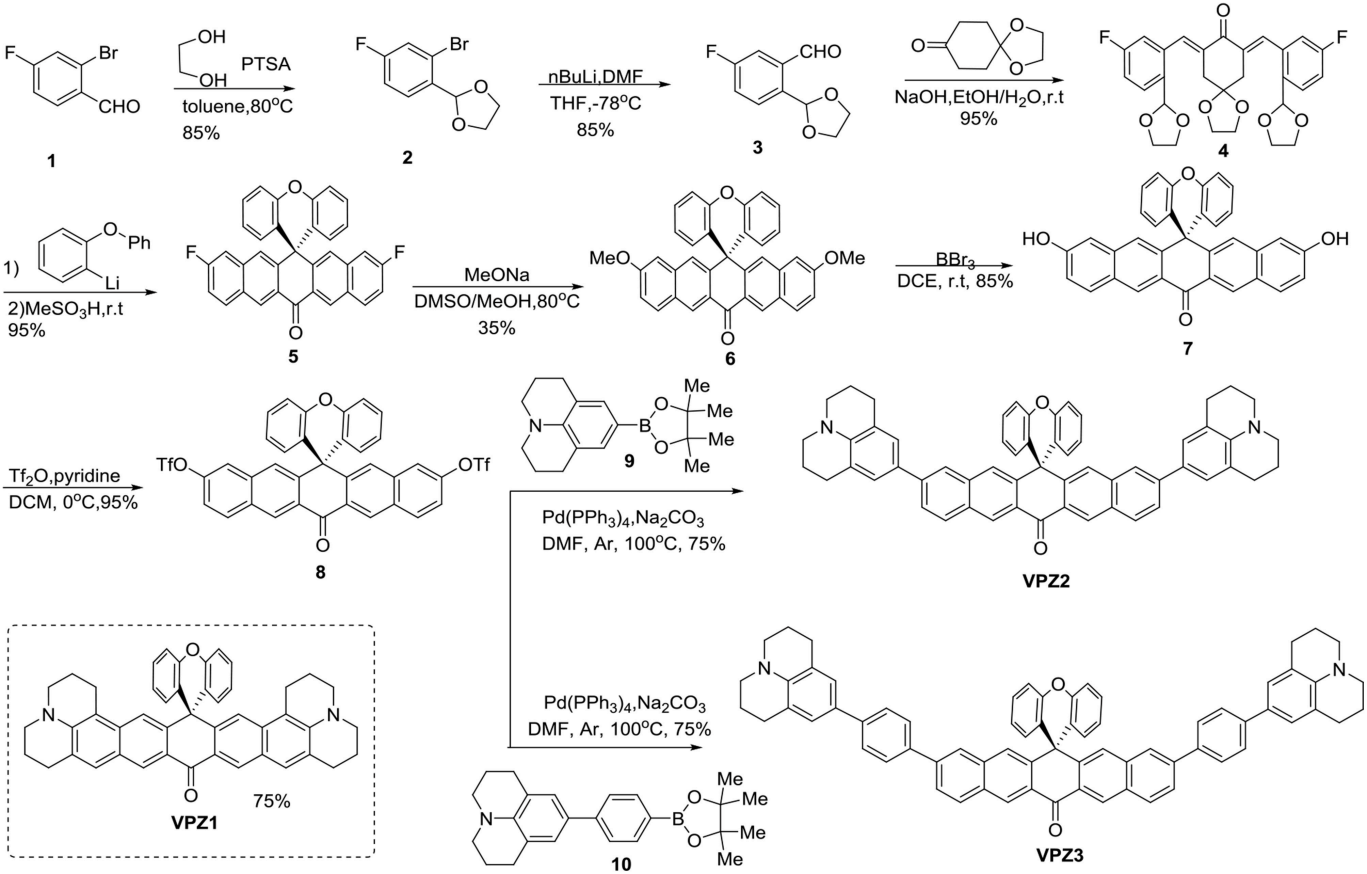
Design and synthesis of the viscosity probe VPZ2 and VPZ3.

**VPZ1** is a known compound and synthesized according to the reported method (Lei et al., 2017). The synthesis **VPZ2** and **VPZ3** are displayed in Scheme 1 2-Bromo-4-fluorobenzaldehyde (**1**) was condensed with ethylene glycol in the presence of p-toluene-sulfonic acid to the corresponding 1,3-dioxolane derivative (**2**) in an 85% yield. Then **2** was treated with nBuLi at −78°C to generate the nucleophilic intermediate phenyllithium reagent *in situ*, which was quenched with DMF to yield the benzaldehyde derivative (**3**) in good yield. Through aldol condensation of **3** and 1,4-cyclohexanedione monoacetal under basic conditions, **4** was obtained in an 95% yield. (2-Phenoxyphenyl) lithium was added into a solution of 4 in THF. The resulting carbinol product was isolated and treated with methylsulfonic acid without further purification to afford **5** through a cascade of reactions. The crystal structure of the compound 5 was obtained and the diphenylether moiety and the bottom dinaphthylmethanone unit are perpendicular. Replacement of fluorine atoms of **5** with methoxide gave **6** in an 35% yield. Then the two methoxys groups of **6** was demethylated by BBr_3_ to get **7**, the hydroxyls of which were converted to triflate by treatment of triflic anhydride to obtain the key intermediate **8**. The viscosity probes (**VPZ2** and **VPZ3**) were furnished by the Suzuki–Miyaura cross coupling reactions with **9** and **10**, respectively. The NMR and the HRMS spectra of all new compounds are provided in the SI (Figures S1–S25). The crystal structure of compound 5 was obtained (Figure S26).

### Spectral Titrations

**Figure 2:**
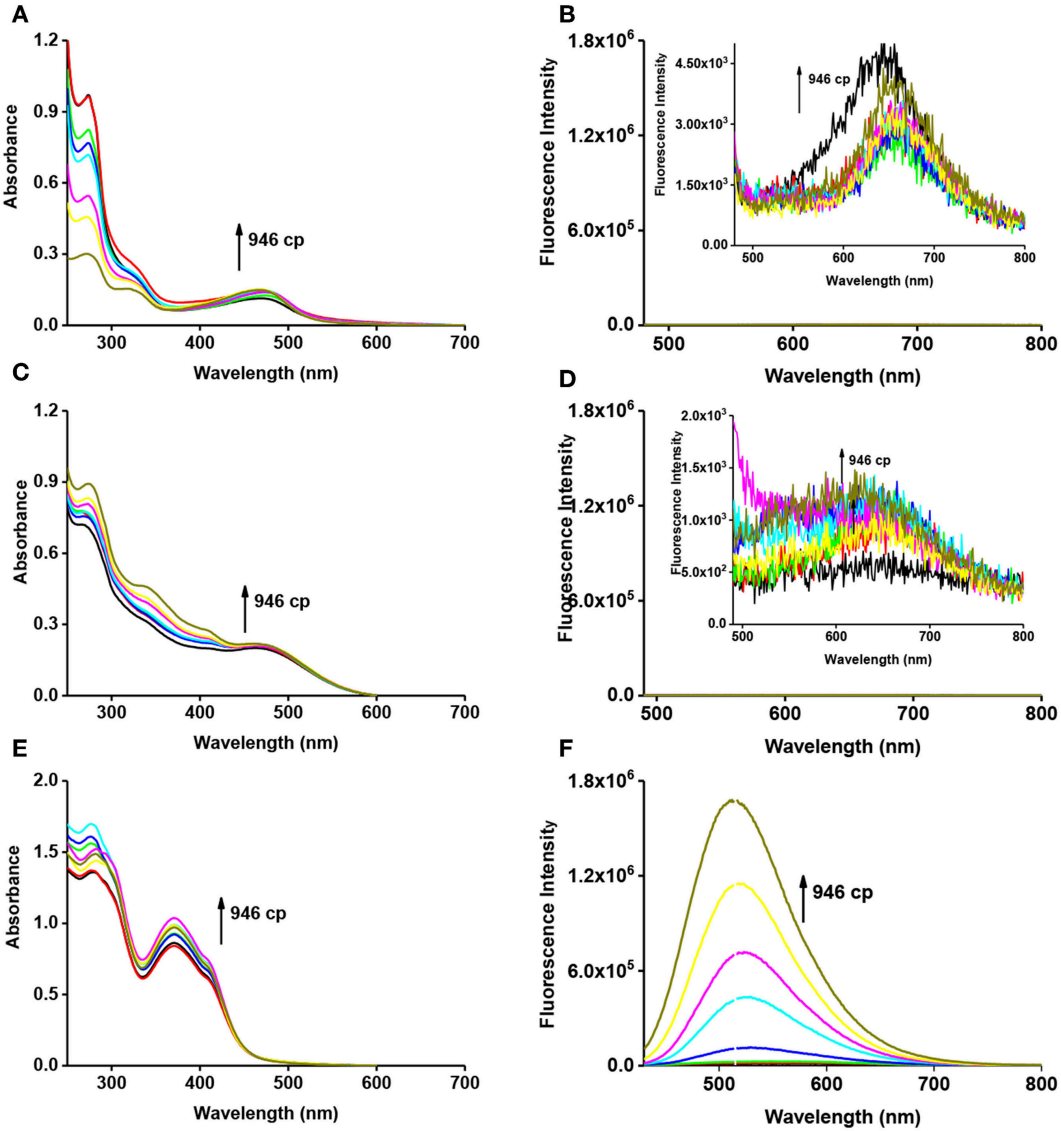
Absorption (**A**, **VPZ1**; **C**, **VPZ2**; **E**, **VPZ3**) and emission (**B**, **VPZ1**; **D**, **VPZ2**; **F**, **VPZ3**) spectra of **VPZ** probes in different viscosity (glycerol and water), concentration: 10 μM.

**Figure 3:**
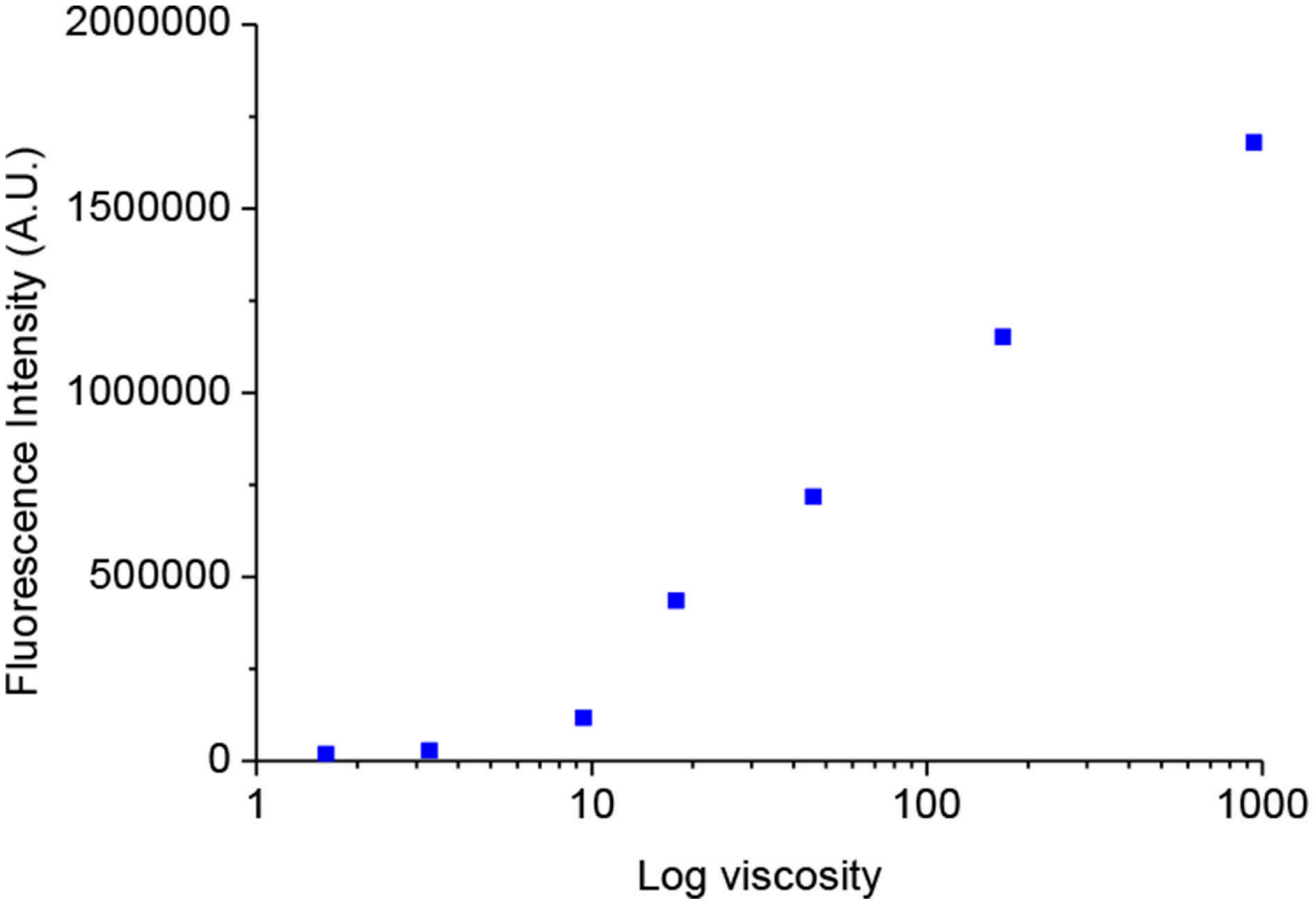
The relationship of the fluorescence intensity of **VPZ3** at varying solvent viscosity.

With the probes in hand, we firstly tested the photophysical properties of these probes (Figure 2, glycerol and water). The maximum absorption wavelength of **VPZ1**, **VPZ2**, and **VPZ3** in pure water is about 470, 465, and 373 nm, respectively. Their fluorescence is very weak but still observable. The emission wavelength of **VPZ1**, **VPZ2**, and **VPZ3** is about 650, 650, and 515 nm, respectively. With their symmetric D-A-D structure, the **VPZ** probes typically exhibit a Stokes shift of ca. 140–190 nm, much larger than that of a list of common fluorophores, such as fluorescein (24 nm), tetramethylrhodamine (25 nm), BODIPY (20 nm), Cy5 (20 nm), Cy7 (23 nm), which is desirable for imaging-based applications. We tested the viscosity related spectral responses (Figure 2). **VPZ** probes were added into solvent mixtures of water and glycerol exhibiting different viscosity values, their absorption and emission spectra were recorded. As shown in Figure 2, the absorption of **VPZ** probes exhibited only very subtle changes with respect to the increase of the solvent viscosity. The fluorescence emission intensity of **VPZ1** and **VPZ2** remained essentially unchanged with respect to the increase of the solvent viscosity. Notably, **VPZ3** show a strong increase in fluorescence (ca.126-fold) with the increase of viscosity. Very interestingly, the fluorescence intensity of **VPZ3** did not immediately increase when the solvent viscosity increased (Figure 3). Instead, the fluorescence intensity remained unchanged until the Log(viscosity) of the solution was higher than 10. After that, the fluorescence of **VPZ3** enhanced linearly with respect to Log(viscosity). This is in good agreement with the design rational of the **VPZ** series of probes. The fluorescence life-time decay of VPZ3 in different solvent mixtures of H2O and glycerol was acquired (Figure S27). Since **VPZ3** has the best performance against different viscosity, we chosen **VPZ3** as the viscosity probe for the cell imaging study. The fact that **VPZ1** and **VPZ2** do not fluoresce in this solvent mixture is intriguing and subject to further in-depth photophysical studies.

### Cell Images

**Figure 4:**
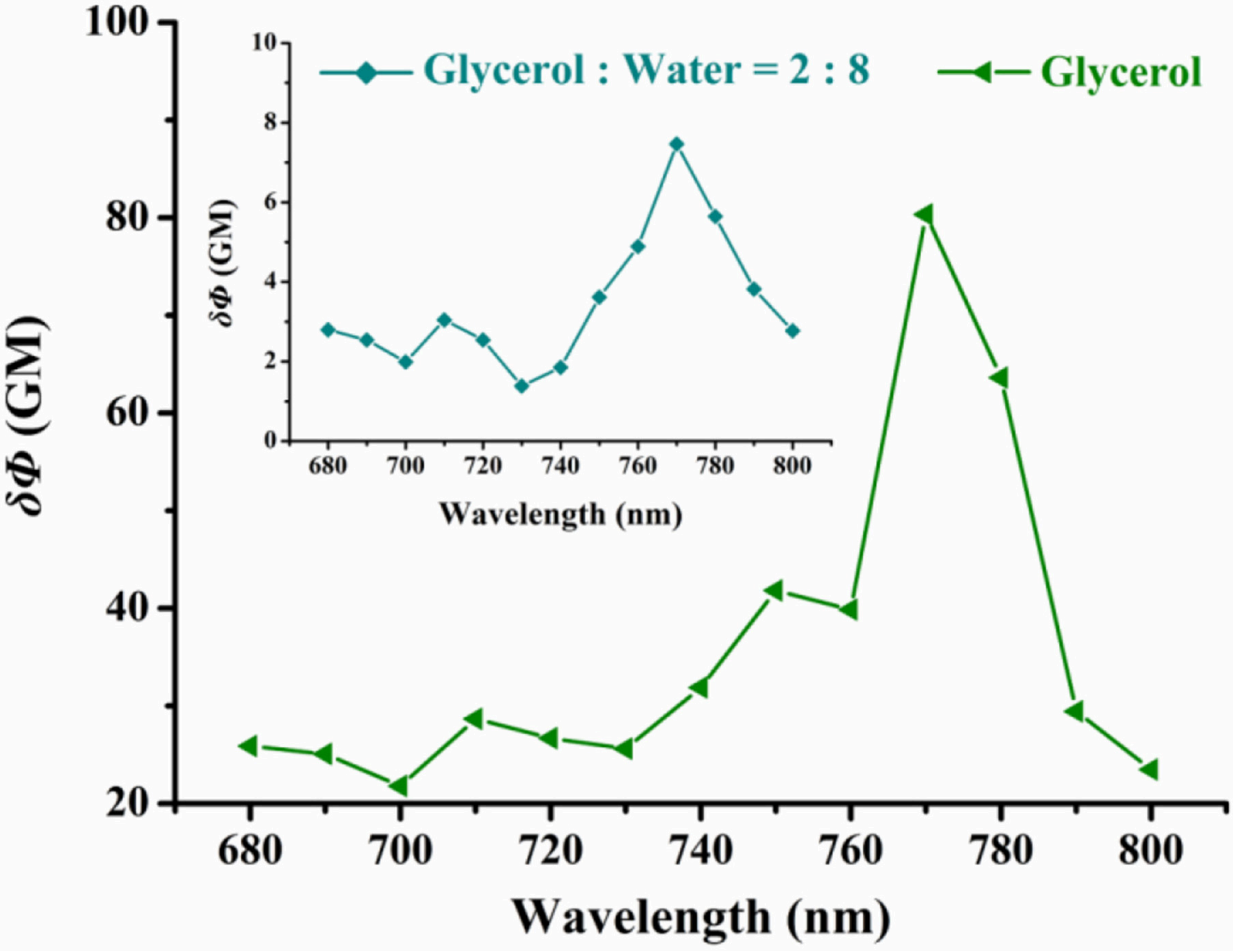
Two-photon excitation action cross-section of **VPZ3** in the glycerol–water system with different viscosities (20% glycerol and 100% glycerol). Two-photon excitation wavelength range: 680–880 nm (Δλ = 10 nm).

The short absorption wavelength of **VPZ3** limits its usage in imaging-based application *in vivo* with one photon excitation. To apply **VPZ3** in cell study, we firstly performed the two-photon cross-section (δ) test (Figure 4) since the short one photon excitation problem can be circumvented by two-photon excitation. The maximum δ is about 80 GM at 770 nm in glycerol. Therefore, 770 nm was used for the cell imaging study.

**Figure 5:**
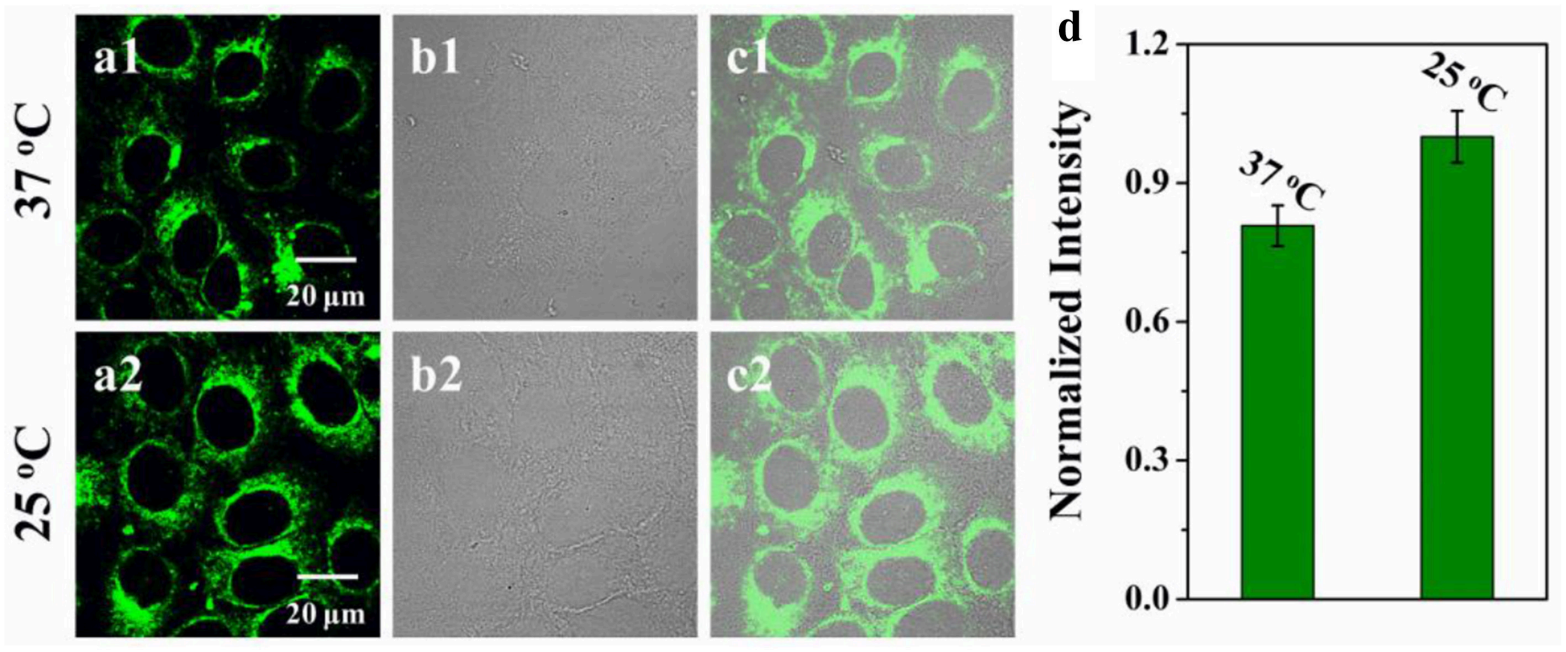
Two-photon confocal images of HepG2 cells incubated with **VPZ3** (10 μM) after 30 min of incubation at **(a1)** 37°C and **(a2)** 25°C, respectively and then washed with 10 mM PBS buffer. λ_ex_ = 770 nm, emission wavelength from 500 to 540 nm; **(b1,b2)** bright-field images of HepG2 cells; **(c1)** the overlay of panels **(a1,b1)**; **(c2)** the overlay of **(a2,b2)**. **(d)** Normalized intensity analysis of the probe at 37 and 25°C, respectively. Scale bars: 20 μm.

The cytotoxicity of **VPZ3** was determined by MTT. In short, HepG2 cells were incubated with **VPZ3** with different concentration for 24 h. The cell viability remained 85% at up to 25 μM (Figure S28). The low cytotoxicity of **VPZ3** promotes us to further explore the possibility of **VPZ3** as a fluorescence probe for the detection of cell viscosity. In order to demonstrate the imaging ability of **VPZ3** in living cells, the HepG2 cells were incubated with **VPZ3** (10 μM) at 37 and 25°C for 30 min separately. The ptimages of the cells were collected under two-photon excitation. As we known, lower temperature means higher viscosity for the cells. The fluorescence intensity of cells incubated at 25°C was higher than those at 37°C significantly (Figure 5). The imaging results supported that the **VPZ3** could be used for monitoring the viscosity in the cytoplasm of living cells.

**Figure 6:**
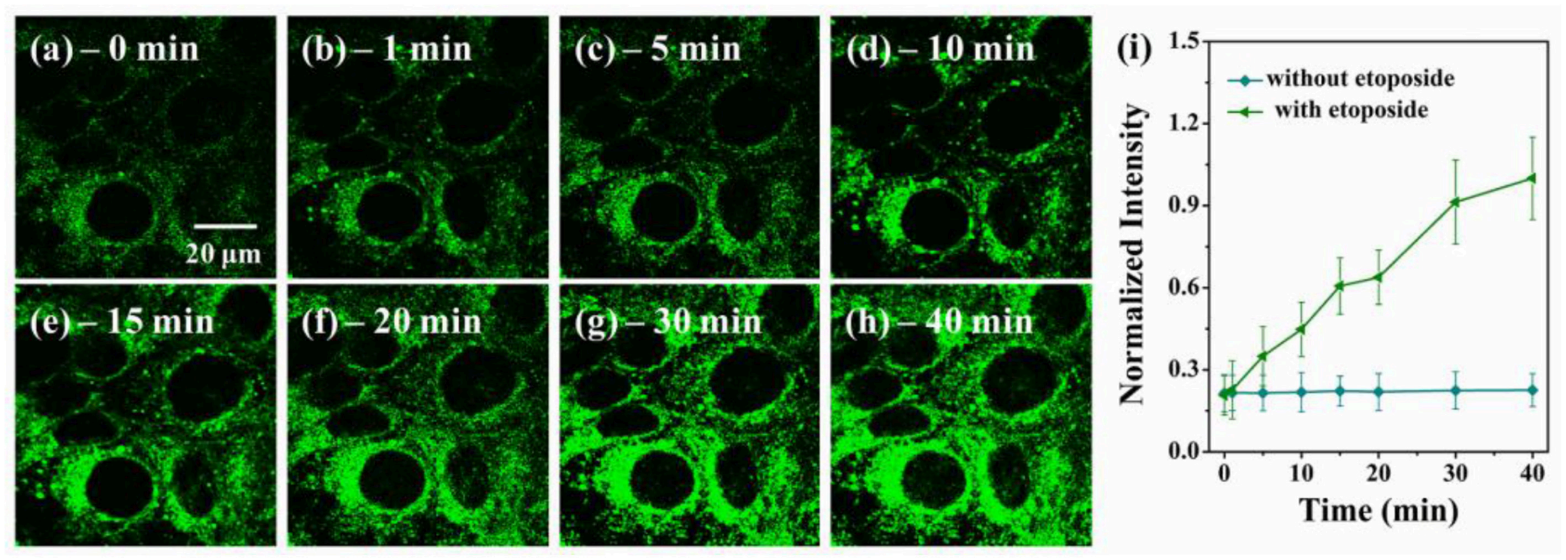
**(a–h)** Two-photon confocal images of HepG2 cells incubated with **VP3** (10 μM) at different time points after the addition of 2.0 μM etoposide, λ_ex_ = 770 nm, emission wavelength from 500 to 540 nm. **(i)** Plot of normalized intensities of **the probe** against time with (triangle, green line) and without (circle, blue line) etoposide. Scale bars: 20 μm.

To further demonstrate the potentials of **VPZ3** in monitoring the micro-viscosity change of cells, **VPZ3** was used to monitor the real-time viscosity change during cell apoptosis. Because etoposide (a chemotherapy drug used to treat many types of cancer) can cause cell death, the micro-viscosity of the cells will change greatly during the apoptosis process. HepG2 cells were incubated with etoposide. The two-photon fluorescence images were collected at different times during the apoptosis process. As shown in Figure 6, the fluorescence intensity of the cells increased greatly during the apoptosis upon addition of etoposide. In contrast, the fluorescence of the cells without the addition of etoposide kept unchanged (Figure S29). These results clearly show that **VPZ3** could be used to monitor the viscosity changes during the apoptosis process.

## Conclusion

In summary, we have developed a series of new fluorescence probes (**VPZ**) for viscosity monitoring based on a symmetric D-A-D framework. Among the three **VPZ** probes, **VPZ3** showed a great “off-on” fluorescence response (ca. 126-fold) with increasing viscosity in the glycerol-water system. **VPZ3** shows low cytotoxicity and could be used to monitor the real-time viscosity change during the cell apoptosis process. We expect **VPZ3** to find applications in basic biological studies related cell apoptosis and disease diagnostics.

## Data Availability

All datasets generated for this study are included in the manuscript and/or the Supplementary Files.

## Author Contributions

ZL prepared the compound synthesis. KX and SQ involved in the synthesis and carried spectral titrations. LH carried out biological studies. YY and XM conceived the project. Everyone contributed to manuscript preparation.

### Conflict of Interest Statement

The authors declare that the research was conducted in the absence of any commercial or financial relationships that could be construed as a potential conflict of interest.
