# Supplementary material for: A Threshold-Limited Fluorescence Probe for Viscosity
Source: Front Chem. 2019 May 14;7:342. doi: 10.3389/fchem.2019.00342 (PMC6527809; doi:10.3389/fchem.2019.00342)
Supplement: Supplementary file 1 [file Data_Sheet_1.docx]

Supplementary Material for

A Threshold-Limited Fluorescence Probes for Viscosity

Zuhai Lei^1,3^, Kai Xin^1^, Shaobing Qiu^1^, Xiangming Meng*^,4^, Youjun Yang*^,1^

^1^ State Key Laboratory of Bioreactor Engineering, Shanghai Key Laboratory of Chemical Biology, School of Pharmacy, East China University of Science and Technology, Meilong Road 130, Shanghai, 200237, P. R. China.

^2^ Department of Chemistry, Shanghai Key Laboratory of Molecular Catalysis and Innovative Materials, State Key Laboratory of Molecular Engineering of Polymers and iChem, Fudan University, Shanghai 200433, P. R. China.

^3^ Department of Chemistry, Anhui University, Hefei, China .

*** Correspondence:**Corresponding Author
Youjun Yang, [youjunyang@ecust.edu.cn](mailto:youjunyang@ecust.edu.cn);
Xiangming Meng, [mengxm@ahu.edu.cn](mailto:mengxm@ahu.edu.cn).

# General methods

# Chemicals were purchased from major venders based in China and used as received without further purification. Analytical grade solvents including petroleum Ether, CH_2_Cl_2_, and EtOAc were purchased from Titan Scientific. Solvents for reactions were dried over solvent stills, i.e. CaH_2_ for CH_2_Cl_2_, sodium/benzophenone for THF, and activated 4A molecular sieves for DMF.

# The 1-D ^1^H-NMR and ^13^C-NMR spectra were acquired over a Bruker AV-400 spectrometer. The 2-D COSY and ROESY spectra were acquired over a Bruker AMX 400 instrument. Chemicals shifts are referenced to the residue solvent peaks and given in ppm. HRMS are acquired on a Micromass GCT spectrometer.

# UV-Vis absorption spectra were acquired over a SHIMADZU UV-2600 UV-vis spectrophotometer. Fluorescence emission spectra were acquired on a PTI-QM4 steady-stead fluorimeter with a 75 W Xenon arc-lamp and a R928 PMT and an InGaAs photodetector. The excitation and emission slits were set to 2 nm.

Measurement of two-photon absorption cross-section (δ)

Two-photon excitation fluorescence (TPEF) spectra were measured using femtosecond laser pulse and Ti: sapphire system (680~1080 nm, 80 MHz, 140 fs, Chameleon II) as the light source. All measurements were carried out in air at room temperature. Two-photon absorption cross-sections were measured using two-photon-induced fluorescence measurement technique. The Two-photon absorption cross sections (*δ*) are determined by comparing their TPEF to that of fluorescein in different solvents, according to the following equation:

$\text{δ=}\text{δ}_{\text{ref}}\frac{\text{F}}{\text{F}_{\text{ref}}}\frac{\text{Φ}_{\text{ref}}}{\text{Φ}}\frac{\text{c}_{\text{ref}}}{\text{c}}\frac{\text{n}_{\text{ref}}}{\text{n}}$ (1)

Here, the subscripts ref stands for the reference molecule. *δ* is the TPA cross-section value, *c* is the concentration of solution, *n* is the refractive index of the solution (because of the low concentrations of the solutions, the refractive indices of the solutions were replaced with those of the solvents), *F* is the TPEF integral intensities of the solution emitted at the exciting wavelength, and *Φ* is the fluorescence quantum yield. The *δ_ref_* value of reference was taken from the literature. (C. Xu, W. W. Webb, ***J. Opt. Soc. Am. B***, 1996, 13, 481-491.)

Cytotoxicity assays

MTT (5-dimethylthiazol-2-yl-2,5-diphenyltetrazolium bromide) assay was performed as previously reported to test the cytotoxic effect of the probe in cells. HeLa cells were passed and plated to ca. 70% confluence in 96-well plates 24 h before treatment. Prior to **the probe** treatment, DMEM (Dulbecco’s Modified Eagle Medium) with 10% FBS (Fetal Bovine Serum) was removed and replaced with fresh DMEM, and aliquots of **the probe** stock solutions (1 mM DMSO) were added to obtain final concentrations of 0, 5, 10, 15, 20 and 25 μM respectively. The treated cells were incubated for 24 h at 37 ^o^C under 5% CO_2_. Subsequently, cells were treated with 5 mg/mL MTT (40 μL/well) and incubated for an additional 4 h (37 ^o^C, 5% CO_2_). Then the cells were dissolved in DMSO (150 μL/well), and the absorbance at 570 nm was recorded. The cell viability (%) was calculated according to the following equation:

$\text{Cell viability \%=}\text{OD}_{\text{570}}\text{(sample)/}\text{OD}_{\text{570}}\text{(control)×100}$ (2)

where OD_570_(sample) represents the optical density of the wells treated with various concentration of **the probe** and OD_570_(control) represents that of the wells treated with DMEM containing 10% FBS. The percent of cell survival values is relative to untreated control cells.

Cell culture and two-photon fluorescence microscopy imaging

For two-photon bio-imaging, HeLa cells were cultured in DMEM supplemented with 10% FBS, penicillin (100 μg/mL), and streptomycin (100 μg/mL) at 37 ^o^C in a humidified atmosphere with 5% CO_2_ and 95% air. Cytotoxicity assays show that **the probe** is safe enough for two-photon bio-imaging at low concentrations, and HeLa cells were cultured and stained with **the probe** (10 μM) within 30 min and washed by PBS buffer. Cells imaging was carried out on a confocal microscope (Zeiss LSM 710 Meta NLO). Two-photon fluorescence microscopy images of labeled cells were obtained by exciting the probe with a mode-locked titanium–sapphire laser source set at wavelength 770 nm.

**Synthesis**

**2-(2-bromo-4-fluorophenyl)-1,3-dioxolane (2)** To a solution of 2-bromo-4-fluorobenzaldehyde (5 g, 23.25 mmol, 1 eq) in 100 mL dry toluene was added ethylene glycol (1.6 g, 25.57 mmol, 1.1 eq), p-toluenesulfonic acid (400 mg, 2.33 mmol, 0.1 eq). The mixture was refluxed for 4 h, a water separator was used to separate the water produced by the reaction. After the the solvent was removed, the mixture quenched by Saturated sodium bicarbonate solution. The crude product was extracted with CH_2_Cl_2_, dried with anhydrous MgSO_4_ and purified by column chromatography with petroleum ether and ethyl acetate (100:2, v/v) as an eluent to afford an oil **2-(2-bromo-4-fluorophenyl)-1,3-dioxolane (2)** (5.1 g) in an 85% yield. ^1^H-NMR (400 MHz, CDCl_3_) δ 7.56-7.52 (m, 1H), 7.29-7.26 (m, 1H), 7.04-6.98 (m, `H), 6.00 (s, 1H), 4.07-3.96 (m, 4H); ^19^F-NMR (376 MHz, CDCl_3_) δ -110.40 (t, *J* = 11.28 Hz); ^13^C-NMR (101 MHz, CDCl_3_) δ 163.8, 161.4, 132.9, 129.3, 129.2, 123.1, 123.0, 120.2, 119.9, 114.6, 114.4, 102.0, 65.4; HRMS (EI) *m/z* Calcd for C_9_H_8_BrFO_2_ [M]^+^, 247.9671; Found, 247.9676

**2-(1,3-dioxolan-2-yl)-5-fluorobenzaldehyde (3).** 7.7 mL n-BuLi (2.5 M in hexane, 1 eq) was injected into a solution of compound **2** (5 g, 19.3 mmol, 1 eq) in 30 mL dry THF at −78 °C dropwise. After the mixture was stirred for 15 minutes, DMF (1.5 g, 21.2 mmol, 1.1 eq) was added and the temperature was slowly heated to room temperature. The mixture was quenched by saturated ammonium chloride solution. Then the resultant solution was extracted with CH_2_Cl_2_, dried with dry MgSO_4_ , filtered and evaporated under reduced pressure. The crude product was purified by column chromatography with petroleum ether and ethyl acetate (100:10, v/v) as an eluent to afford **2-(1,3-dioxolan-2-yl)-5-fluorobenzaldehyde (3)** (3.42 g) in a 85% yield. ^1^H-NMR (400 MHz, CDCl_3_) δ 10.40 (d, *J* = 2.44 Hz, 1H), 7.71 (q, *J* = 8.56 Hz, 1H), 7.62 (dd, *J* = 8.80 Hz, 2.72Hz, 1H), 7.31-7.26 (m, 1H), 6.30 (s, 1H), 4.15-4.09 (m, 4H); ^19^F-NMR (376 MHz, CDCl_3_) δ -111.10; ^13^C-NMR (101 MHz, CDCl_3_) δ 190.1, 163.5, 135.5, 129.5, 120.4, 120.0, 115.8, 100.9, 65.3; HRMS (EI) *m/z,* Calcd for C_10_H_9_FO_3_ [M]^+^, 196.0536, Found, 196.0537.

**9-bis((E)-2-(1,3-dioxolan-2-yl)-5-fluorobenzylidene)-1,4-dioxaspiro [4.5] decan-8-one (4).** Compound **3** (3 g, 14.41 mmol, 1 eq) and 1,4-dioxaspiro [4.5]decan-8-one (1.13 g, 7.2 mmol, 0.5 eq) were dissolved in 10 ml ethanol , and a 40% NAOH solution was slowly added dropwise. The mixture was stirred for 4 h under room temperature. The precipitate is collected by vacuum filtration and purified by recrystallization in a solution of petroleum ether and ethyl acetate to give a fluffy bright yellow solid compound **4** (3.2 g) in a 95% yield. ^1^H-NMR (400 MHz, CDCl_3_) δ 8.06 (s, 2H), 7.60 (t, *J* = 8.40 Hz, 2H), 7.06 (td, *J* = 8.30 Hz, 2.04 Hz, 2H), 6.93 (td, *J* = 9.32 Hz, 1.84 Hz, 2H), 5.84 (s, 2H), 4.16-4.12 (m, 4H), 4.02-3.99 (m, 4H), 3.87 (s, 4H), 2.98 (s, 4H); ^19^F-NMR (376 MHz, CDCl_3_) δ -112.13 (t, *J* = 15.04 Hz); ^13^C-NMR (101 MHz, CDCl_3_) δ 187.3, 162.7 (*J* = 252 Hz), 137.2, 137.1, 136.5, 134.6, 132.4, 132.3, 129.1, 129.0, 115.8, 115.6, 115.2, 115.0, 106.6, 101.4, 65.4, 64.7, 37.1. HRMS (ESI) *m/z,* Calcd for C_28_H_26_F_2_O_7_ [M+H]^+^, 513.1719, Found, 513.1724.

**3,9-difluoro-13H-spiro[pentacene-6,9'-xanthen]-13-one (5).** A solution of n-BuLi (1.6 M in hexane, 5.5 mL) was injected into a solution of oxydibenzene (1 g, 59 mmol) in dry THF at 0 °C. The mixture was stirred for 2 h under Ar. The resulting solution was added to a dry THF solution containing compound 4 (1 g, 1.5 mmol), and the mixture solution was stirred for another 1 h under temperature. A saturated ammonium chloride solution was poured and the crude product was extracted with CH_2_Cl_2_, purified by column chromatography with petroleum ether and ethyl acetate (100:5, v/v) to afford a yellow solid **5** (0.34 g) in a 35% yield. ^1^H-NMR (400 MHz, CDCl_3_) δ 9.05 (s, 2H), 8.05 (t, *J* = 7.18 Hz, 2H), 7.50 (s, 2H), 7.32-7.18 (m, 8H), 6.78 (t, *J* = 7.46 Hz, 2H), 6.60 (d, *J* = 7.84 Hz, 2H); ^19^F-NMR (376 MHz, CDCl_3_) δ -109.17—109.24 (m); ^13^C-NMR (101 MHz, CDCl_3_) δ 183.9, 162.3 (*J* = 250 Hz), 149.2, 147.9, 137.3, 137.2, 132.4, 132.3, 131.0, 130.9, 130.9, 128.9, 128.7, 128.5, 128.2, 127.6, 127.6, 123.8, 117.9, 117.7, 111.0, 110.8, 46.1. HRMS (ESI) *m/z* Calcd for C_34_H_19_F_2_O_2_ [M+H]^+^, 497.1357, Found, 497.1352.

**3,9-dimethoxy-13H-spiro [pentacene-6,9'-xanthen]-13-one (6).** ^1^H-NMR (400 MHz, CDCl_3_) δ 8.98 (s, 2H), 7.94 (d, *J* = 7.8 Hz, 2H), 7.41 (s, 2H), 7.30 (dd, *J* = 7.8 Hz, 2.4 H, 2H), 7.19 (t, *J* = 8.2 Hz, 2H), 7.12 (dd, *J* = 8.0 Hz, 2.4 Hz, 2H), 6.88 (d, *J* = 2.4 Hz, 2H), 6.78 (t, *J* = 8.4 Hz, 2H), 6.65 (d, *J* = 7.8 Hz, 2H), 3.82 (s, 6H); ^13^C-NMR (101 MHz, CDCl_3_) δ 184.0, 159.8, 149.2, 147.8, 137.9, 131.2, 131.2, 130.1, 129.1, 128.4, 127.9, 127.3, 126.5, 123.7, 120.4, 116.6, 105.11, 7.35, 77.0, 76.7, 55.3, 46.1. HRMS (ESI) *m/z* Calcd for C_36_H_24_O_4_ [M+Na]^+^, 543.1572; Found, 543.1571.

**3,9-dihydroxy-13H-spiro[pentacene-6,9'-xanthen]-13-one (7).** Compound **6** (500 mg, 0.96 mmol) was dissolved in 200 mL of 1,2-dichloroethane. 1 mL of BBr_3_ was added slowly and the mixture was stirred for 3 h under room temperature. The mixture was quenched by 100 mL H_2_O. The resulting solution was extracted with CH_2_Cl_2_, dried by anhydrous Na_2_SO_4_ and concentrated under reduced pressure. The crude product was purified by column chromatography with petroleum ether, dichloromethane and ethyl acetate (100:20:10, v/v/v) to get a yellow solid **7** (402 mg) in a 85% yield. ^1^H-NMR (400 MHz, DMSO) δ 10.28 (s, 2H), 8.87 (s, 2H), 8.08 (d, *J* = 9.04 Hz, 2H), 7.35(d, *J* = 7.56 Hz, 2H), 7.21 (d, *J* = 10.24Hz, 2H), 6.89 (s, 2H), 7.13 (dd, *J* = 8.92 Hz, 2.16Hz, 2H), 6.89 (d, *J* = 1.76 Hz, 2H), 6.82 (t, *J* = 7.08 Hz, 2H); 6.57 (dd, *J* = 7.88 Hz, 1.56Hz, 2H); ^13^C-NMR (101 MHz, DMSO) δ 182.47, 158.28, 148.50, 147.48, 137.69, 131.72, 130.71, 128.94, 128.67, 128.20, 128.15, 126.06, 125.33, 123.86, 120.37, 116.73, 107.91, 45.46.

**13-oxo-13H-spiro[pentacene-6,9'-xanthene]-3,9-diyl bis(trifluoromethanesulfonate) (8).** A miture of compound **7** (1 g, 2.03 mmol), trifluoromethanesulfonic anhydride (1.15 g, 4.06 mmol) and pyridine (481.81 mg, 6.09 mmol) in dichloromethane was stirred for 2h at 0 °C. After the temperature was cooled to room temperature, the mixture solution was quenched by water. The organic layer dried with anhydrous sodium sulfate, filtered and concentrated under reduced pressure. The crude product was purified by column chromatography with petroleum ether, dichloromethane and ethyl acetate (100:10:10, v/v/v) to get a white solid **8** (1.46 g) in a 95% yield. ^1^H-NMR (400 MHz, CDCl_3_) δ 9.11 (s, 2H), 8.15 (d, *J* = 9.04 Hz, 2H), 7.30 (s, 2H), 7.58 (s, 2H), 7.38 (d, *J* = 9.08 Hz, 2H), 7.34 (d, *J* = 8.20 Hz, 2H), 7.22 (d, *J* = 7.40 Hz, 2H), 6.81 (t, *J* = 7.34 Hz, 2H), 6.58 (d, *J* = 7.80 Hz, 2H); ^19^F-NMR (376 MHz, CDCl_3_) δ -72.83. ^13^C-NMR (101 MHz, CDCl_3_) δ 183.6, 149.2, 148.9, 148.5, 136.3, 132.7, 132.1, 130.8, 130.5, 129.0, 128.9, 128.6, 127.8, 124.1, 120.9, 120.3, 119.3, 117.3, 46.3. HRMS (ESI)^+^ *m/z,* Calcd for C_36_H_18_F_6_O_8_S_2_ (M+H)^+^: 757.0426, found: 757.0424.

**3,9-bis(2,3,6,7-tetrahydro-1H,5H-pyrido[3,2,1-ij]quinolin-9-yl)-13H-spiro[pentacene-6,9'-xanthen]-13-one (VP2).** A mixture of compound **8** (100 mg, 0.13 mmol), **9** (55 mg, 0.15 mmol) and sodium carbonate (42 mg, 0.4 mmol) in 30 mL DMF under Ar was stirred for 10 min, then the catalytic equivalent of tetrakistriphenylphosphine palladium was added. The temperature was heated to 100 °C with stirring for 8 h under Ar. After the temperature was cooled to room temperature, DMF was removed under reduced pressure. The mixture was diluted with 100 mL dichloromethane and washed with water (50 mL, 3 times). The organic layer was dried with dry Na_2_SO_4_, filtered and evaporated under reduced pressure. The crude product was purified by column chromatography with petroleum ether, dichloromethane and ethyl acetate (100:40:2, v/v/v) to get a yellow solid **VPZ2** (95 mg) in a 75% yield. ^1^H-NMR (400 MHz, CDCl_3_) δ 9.04 (s, 2H), 8.03 (d, *J* = 9.16 Hz, 2H), 7.73 (d, *J* = 6.16 Hz, 2H), 7.72 (s, 2H), 7.54 (s, 2H), 7.31 (d, *J* = 8.28 Hz, 2H), 7.19 (td, *J* = 7.74 Hz, 1.52 Hz, 2H), 7.14 (s, 4H), 6.78 (t, *J* = 7.48 Hz, 2H), 7.19 (dd, *J* = 7.88 Hz, 1.24 Hz, 2H), 3.18 (t, *J* = 5.56 Hz, 8H), 2.80 (t, *J* = 6.38 Hz, 8H), 2.02-1.96(m, 8H), 1.31 (s, 12H); ^13^C-NMR (101 MHz, CDCl_3_) δ 184.4, 149.3, 147.5, 143.1, 141.5, 137.0, 131.6, 131.3, 130.2, 129.9, 129.3, 128.6, 128.0, 127.7, 126.9, 126.2, 125.9, 123.8, 123.1, 121.8, 116.8, 50.0, 46.3, 27.9, 22.1. HRMS (ESI) ^+^, *m/z,* Calcd for C_58_H_46_N_2_O_2_ (M+H)^+^: 803.3638, found: 803.3638.

**3,9-bis(4-(2,3,6,7-tetrahydro-1H,5H-pyrido[3,2,1-ij]quinolin-9-yl)phenyl)-13H-spiro[pentacene-6,9'-xanthen]-13-one (VPZ3).** A mixture of compound **8** (100 mg, 0.13 mmol), **10** (55 mg, 0.15 mmol) and sodium carbonate (42 mg, 0.4 mmol) in 30 mL DMF under Ar was stirred for 10 min, then the catalytic equivalent of tetrakistriphenylphosphine palladium was added. The temperature was heated to 100 °C with stirring for 8 h under Ar. After the temperature was cooled to room temperature, DMF was removed under reduced pressure. The mixture was diluted with 100 mL dichloromethane and washed with water (50 mL, 3 times). The organic layer was dried with dry Na_2_SO_4_, filtered and evaporated under reduced pressure. The crude product was purified by column chromatography with petroleum ether, dichloromethane and ethyl acetate (100:40:2, v/v/v) to get a yellow solid **VPZ3** (95 mg) in a 75% yield. ^1^H-NMR (400 MHz, CDCl_3_) δ 9.09 (s, 2H), 8.12 (d, *J* = 8.64 Hz, 2H), 7.86 (s, 2H), 7.79 (dd, *J* = 8.52 Hz, 1.52Hz, 2H), 7.65 (d, *J* = 8.36 Hz, 2H), 7.63 (d, *J* = 7.12 Hz, 4H), 7.58 (d, *J* = 8.36 Hz, 4H), 7.31 (d, *J* = 7.56 Hz, 2H), 7.19 (td, *J* = 8.40 Hz, 1.48 Hz, 2H), 7.10 (s, 4H), 6.79 (t, *J* = 7.50 Hz, 2H), 6.68 (dd, *J* = 7.92 Hz, 1.32 Hz, 2H), 3.18 (t, *J* = 5.56 Hz, 8H), 2.83 (t, *J* = 6.38 Hz, 8H), 2.04-1.98(m, 8H), 1.31 (s, 12H); ^13^C-NMR (101 MHz, CDCl_3_) δ 169.35 149.2, 147.5, 142.7, 141.1, 137.3, 131.9, 131.1, 130.9, 130.6, 130.1, 129.0, 128.7, 128.3, 128.1, 128.0, 127.4, 127.4, 126.5, 125.4, 125.0, 124.9, 123.7, 121.8, 116.8, 50.0, 29.7, 27.8, 22.0. HRMS (EI)^+^, *m/z,* Calcd for C_70_H_55_N_2_O_2_ (M+H)^+^: 955.4264, found: 955.4262.


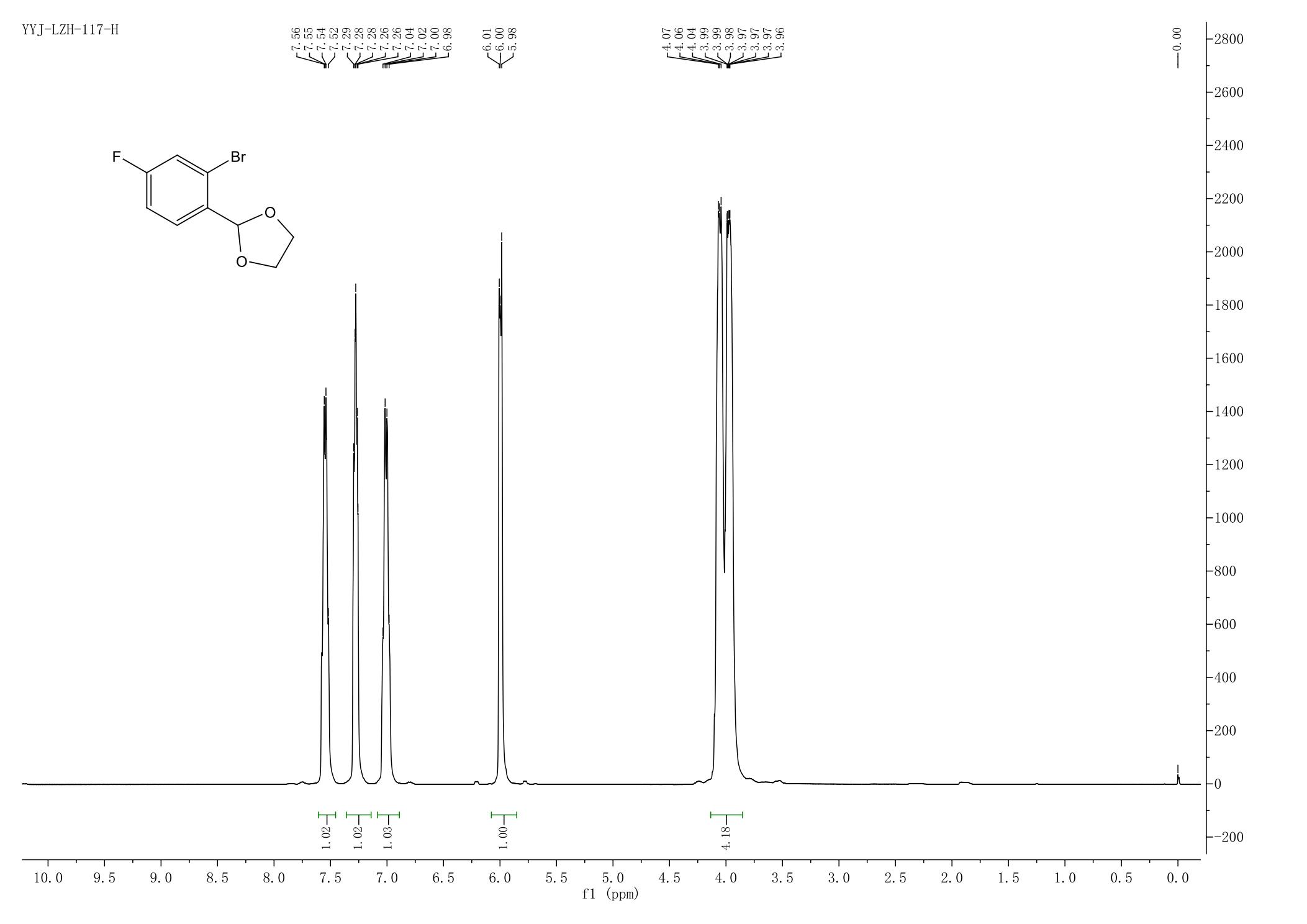


**Fig S1. The ^1^H-NMR of compound 2 in CDCl_3_.**

**
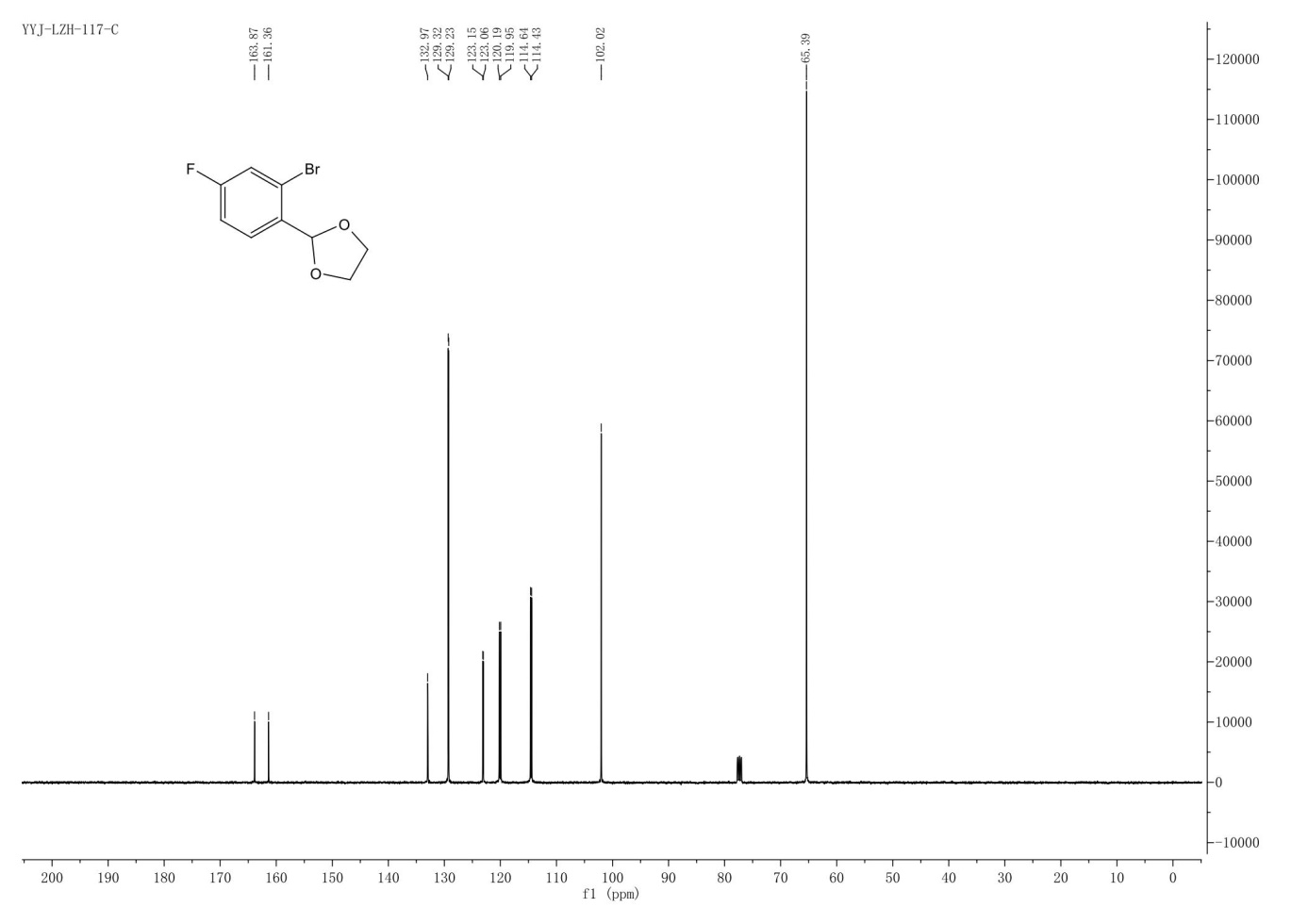
**

**Fig S2. The ^13^C-NMR of compound 2 in CDCl_3._**

**
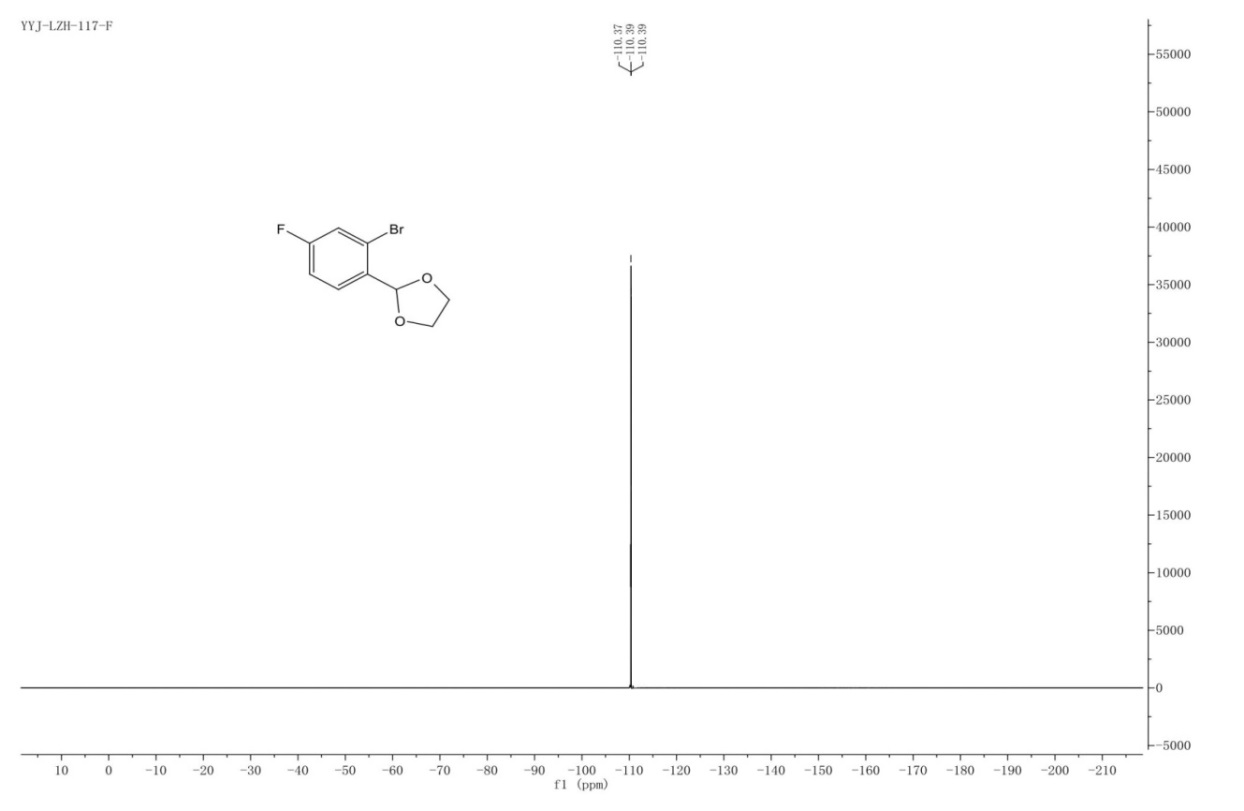
**

**Fig S3. The ^19^F-NMR of compound 2 in CDCl_3._**

**
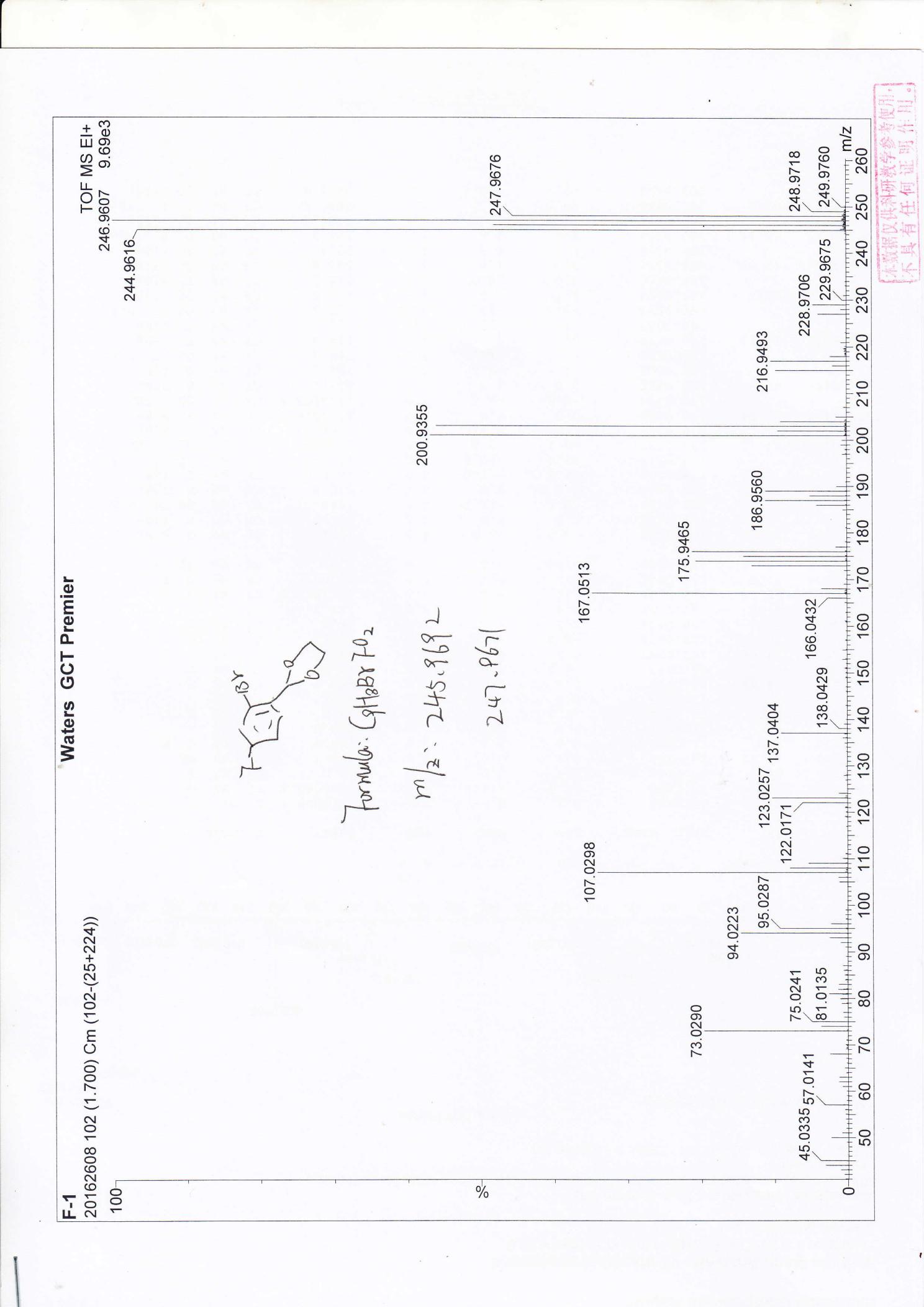
**

**Fig S4. The HR-MS of compound 2.**

**
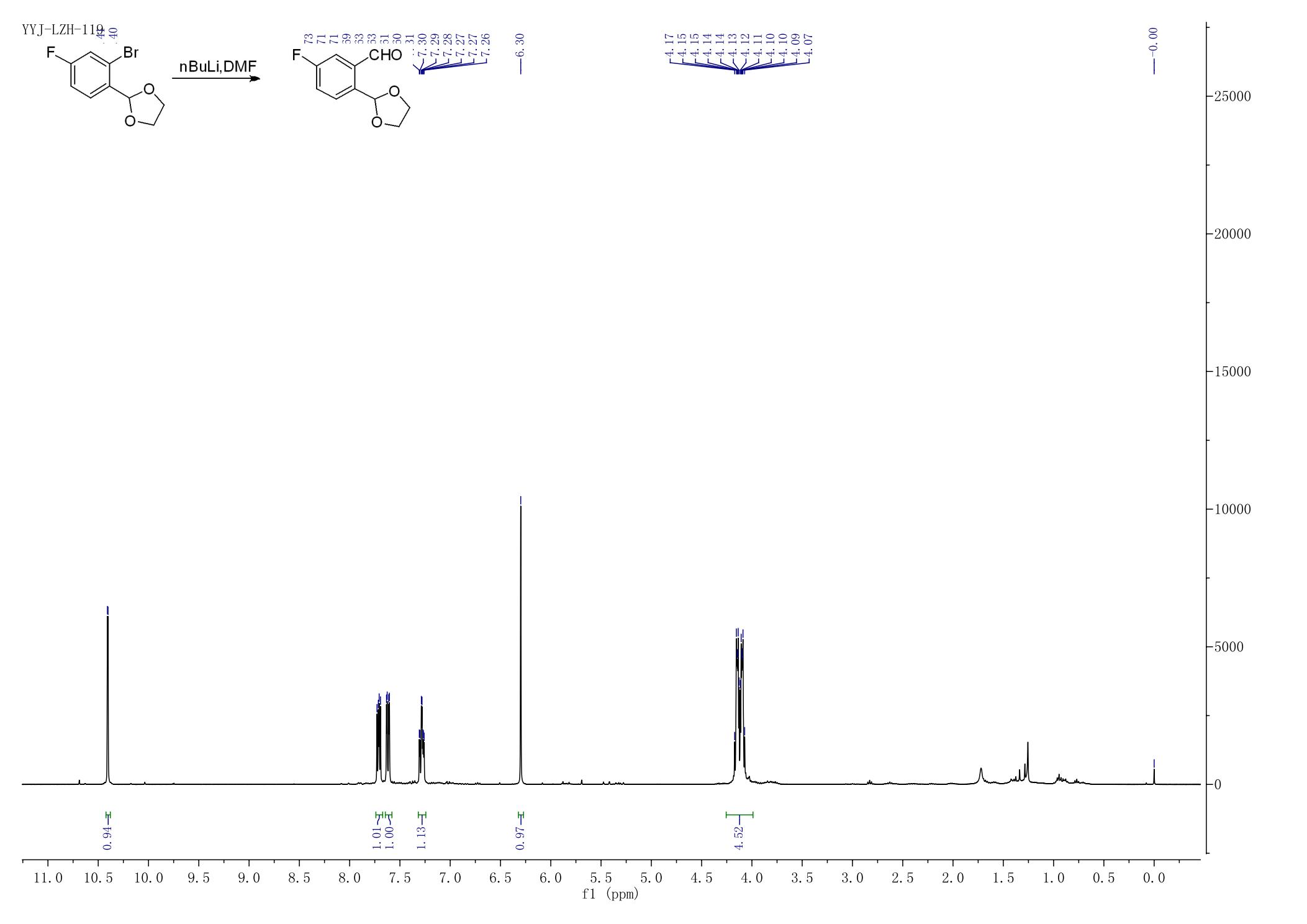
**

**Fig S5. The ^1^H-NMR of compound 3 in CDCl_3_.**

**
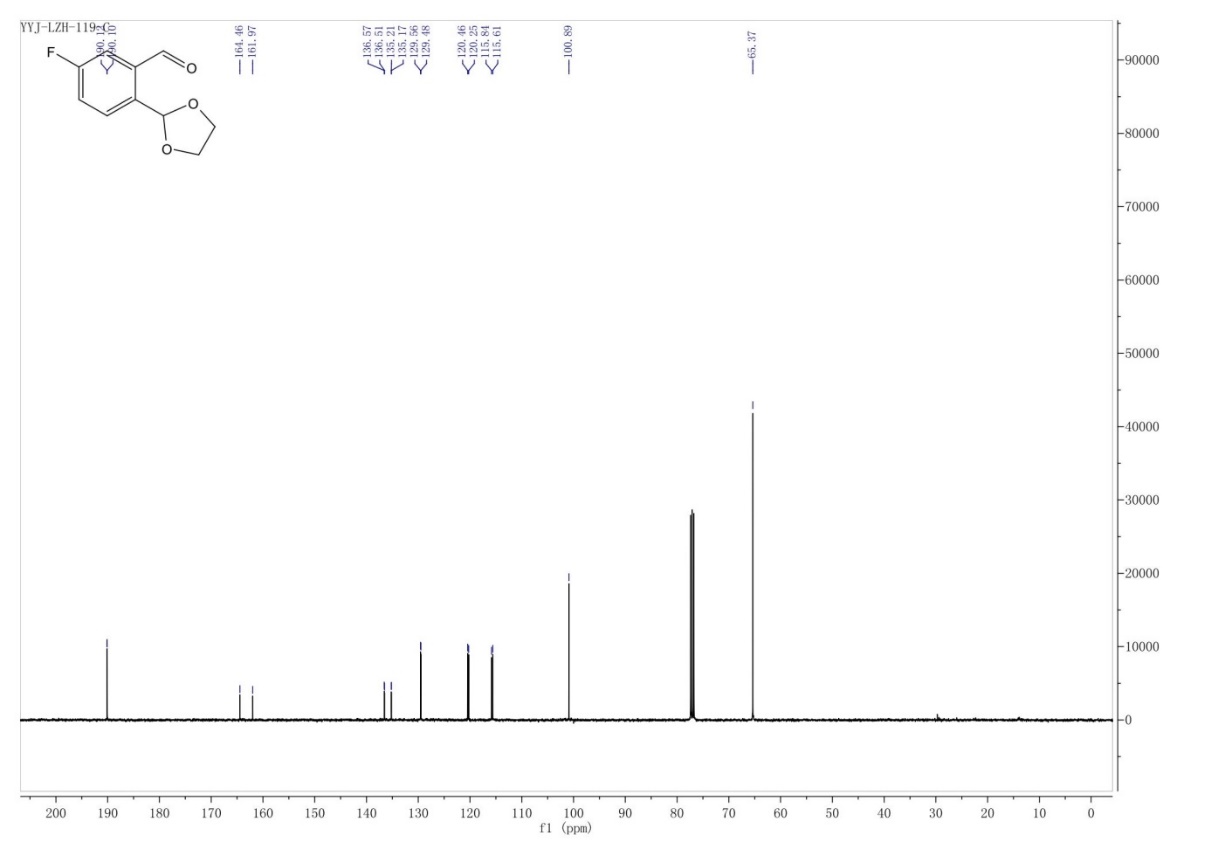
**

**Fig S6. The ^13^C-NMR of compound 3in CDCl_3._**

**
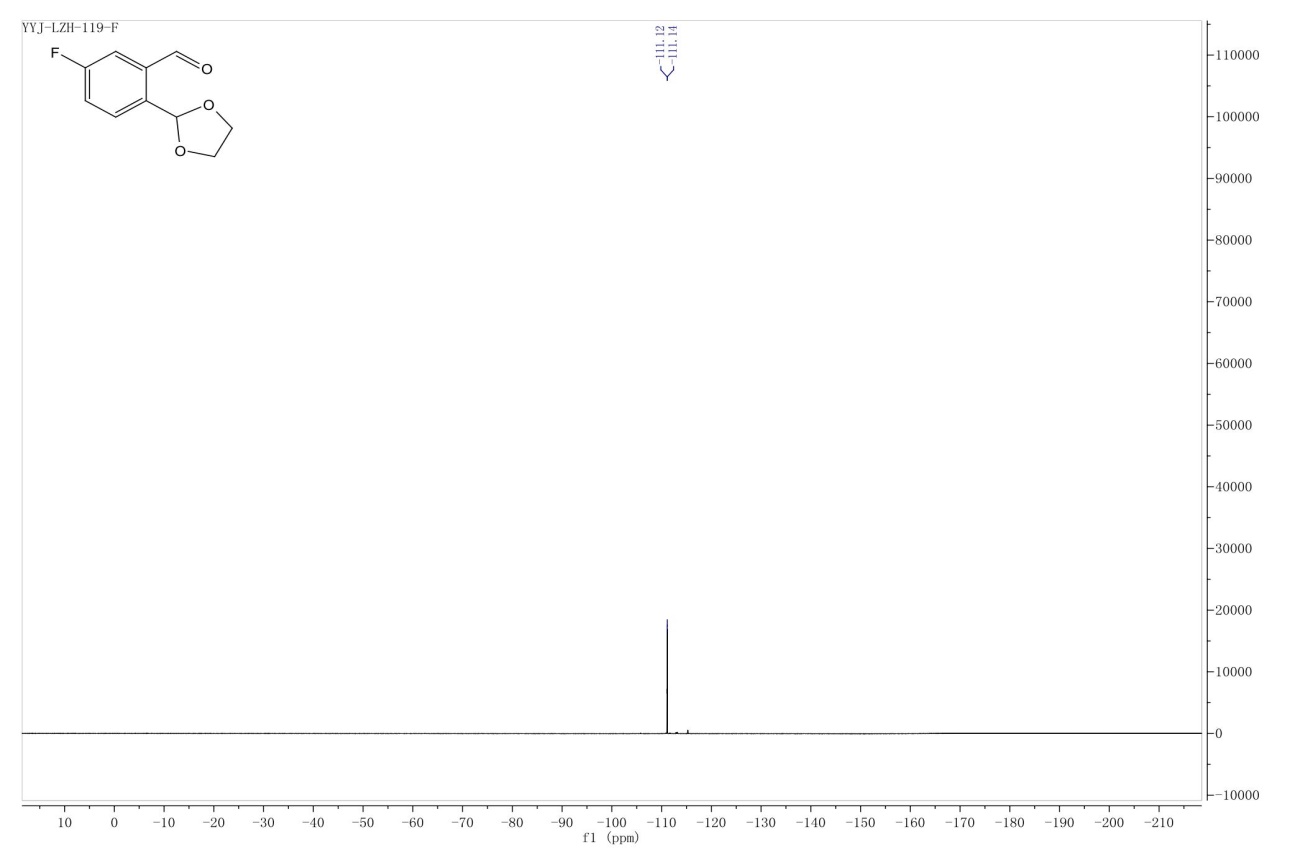
**

**Fig S7. The ^19^F-NMR of compound 3in CDCl_3._**

**_
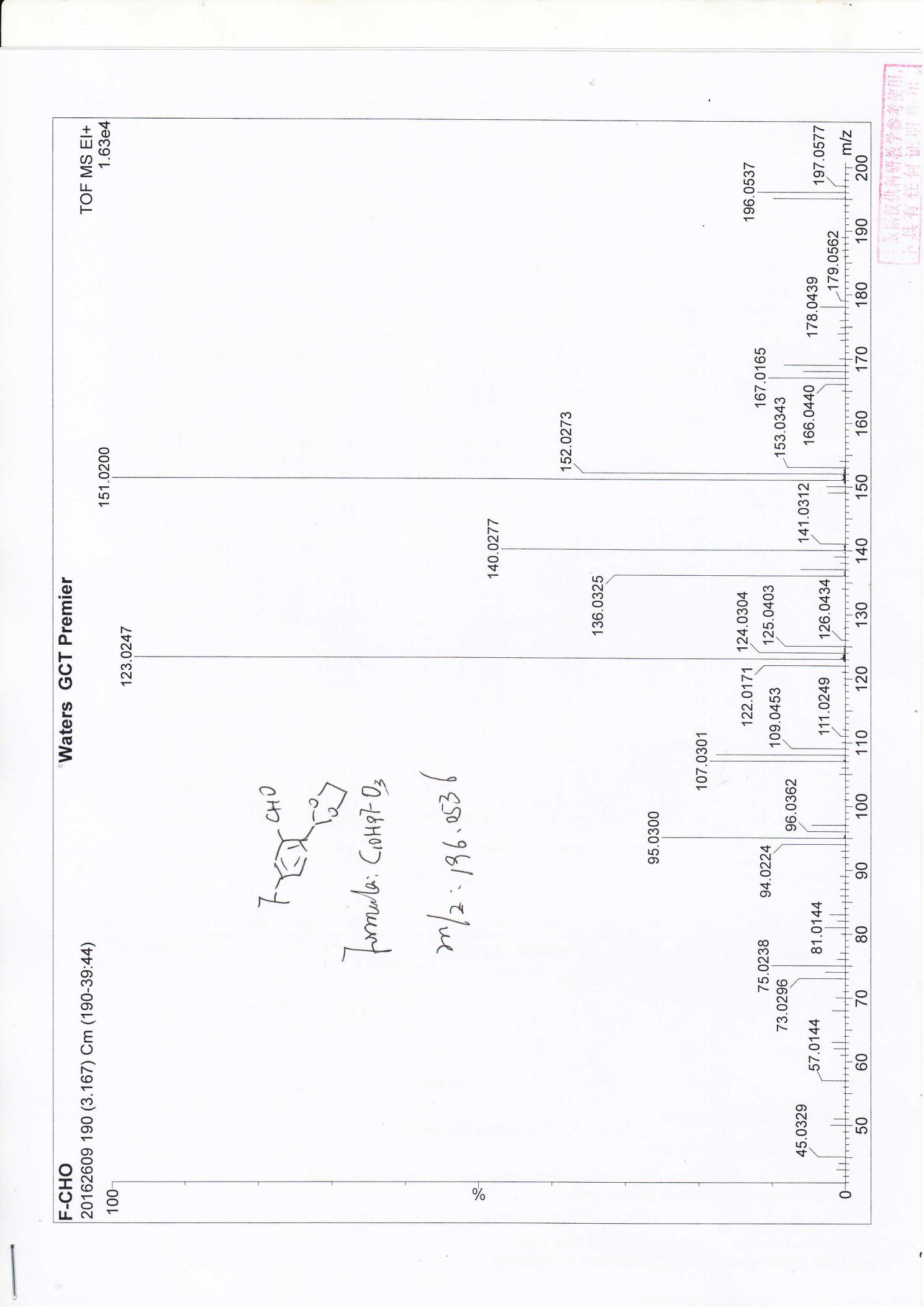
_**

**Fig S8. The HR-MS of compound 3.**

**
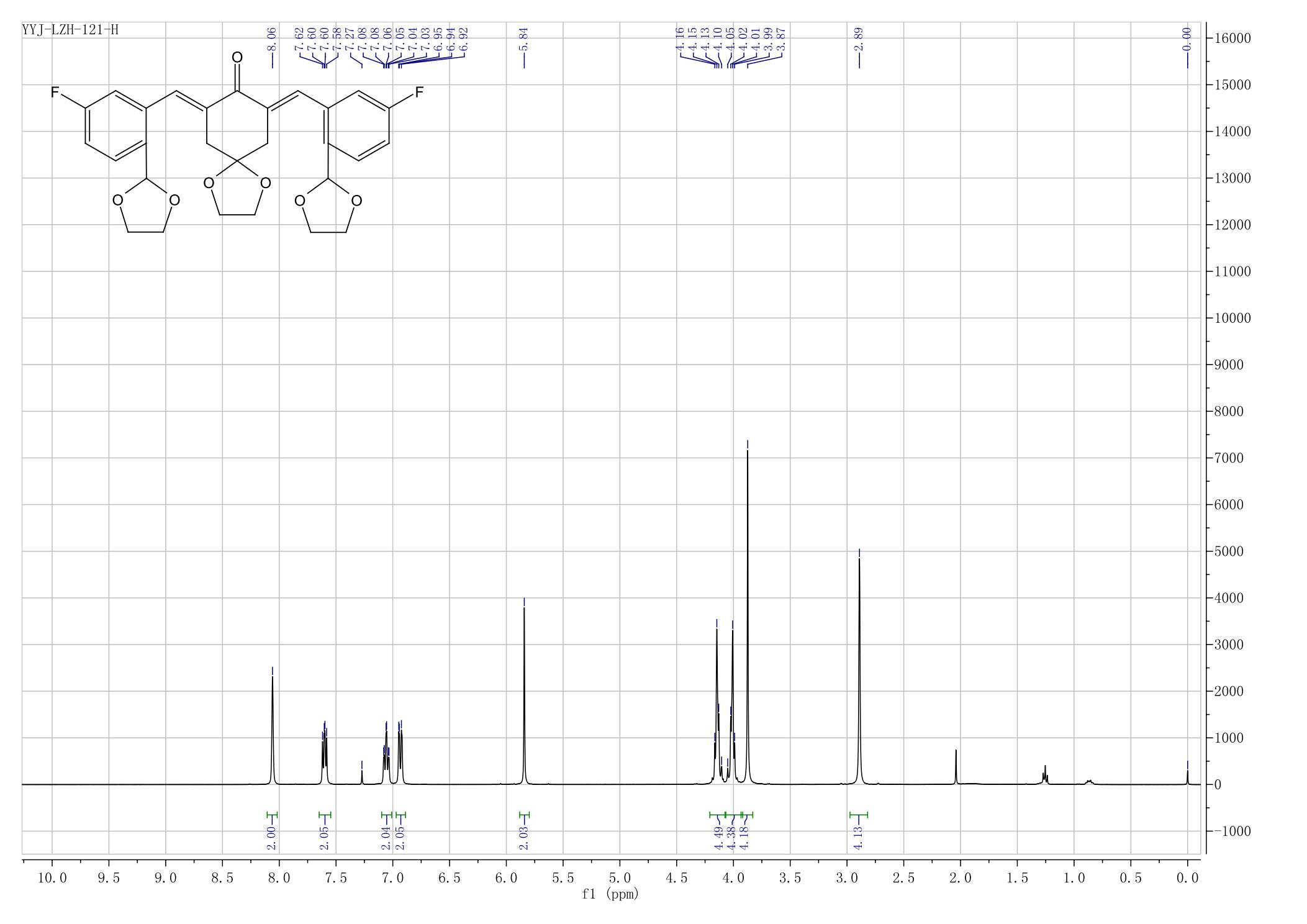
**

**Fig S9. The ^1^H-NMR of compound 4 in CDCl_3_.**

**
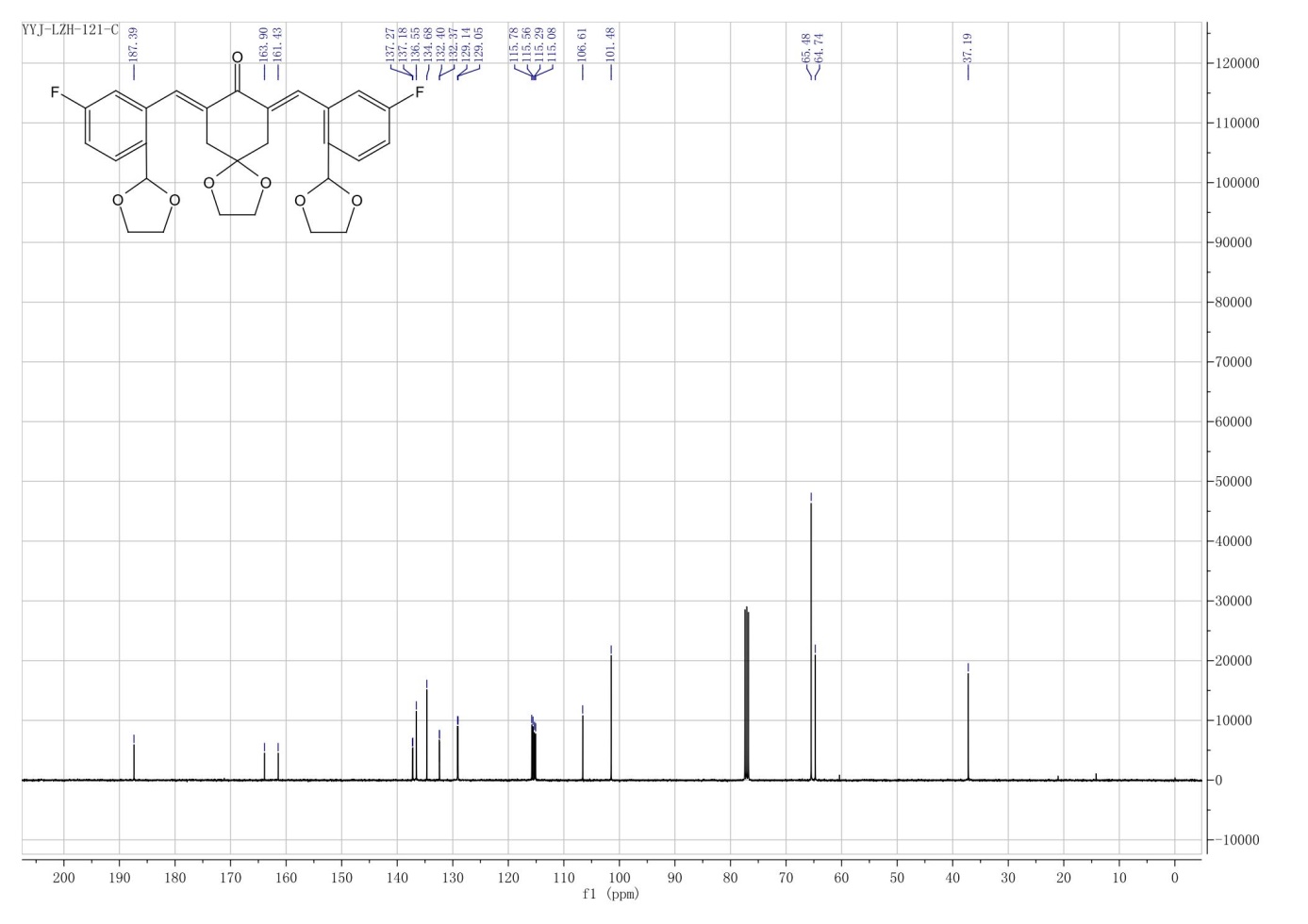
**

**Fig S10. The ^13^C-NMR of compound 4 in CDCl_3._**

**
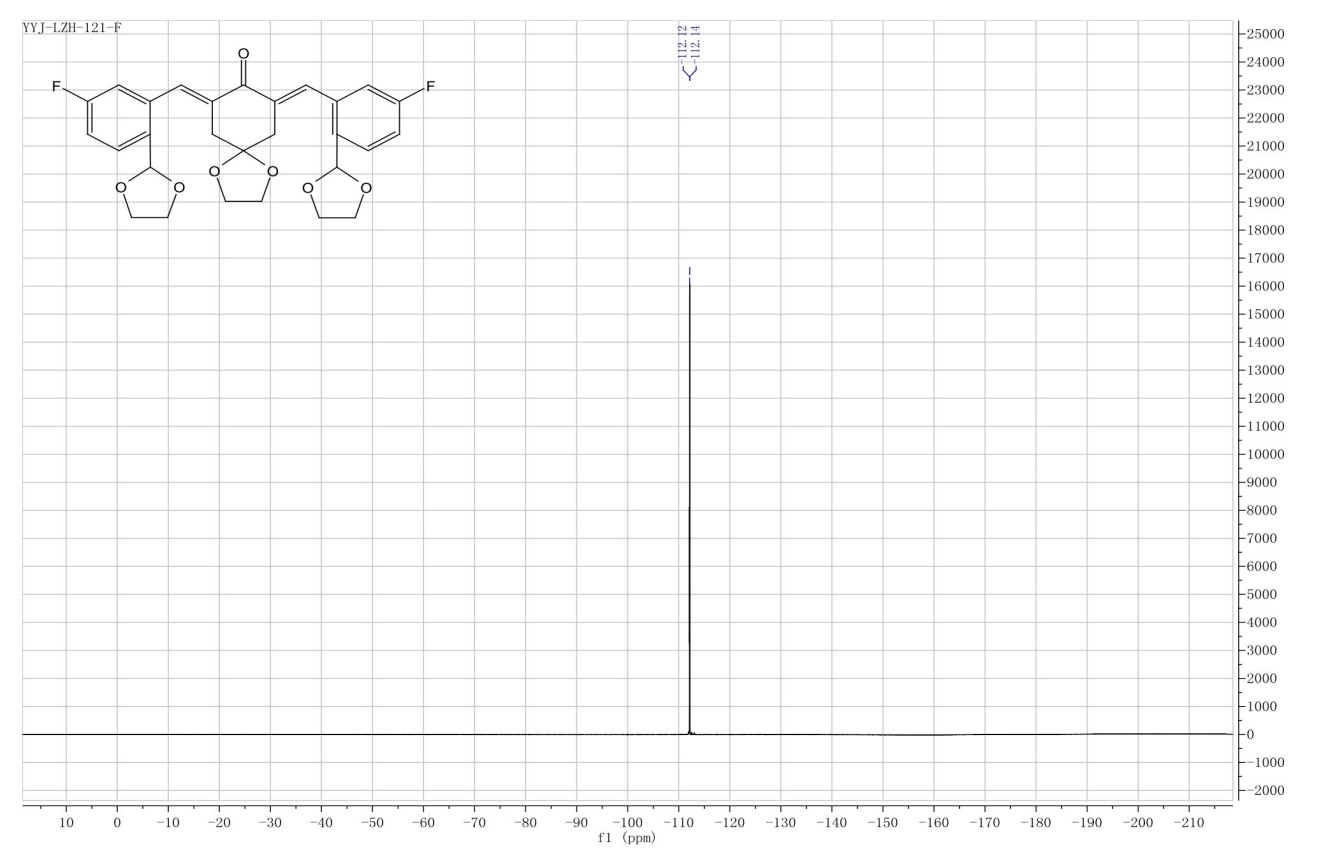
**

**Fig S11. The ^19^F-NMR of compound 4 in CDCl_3._**

**_
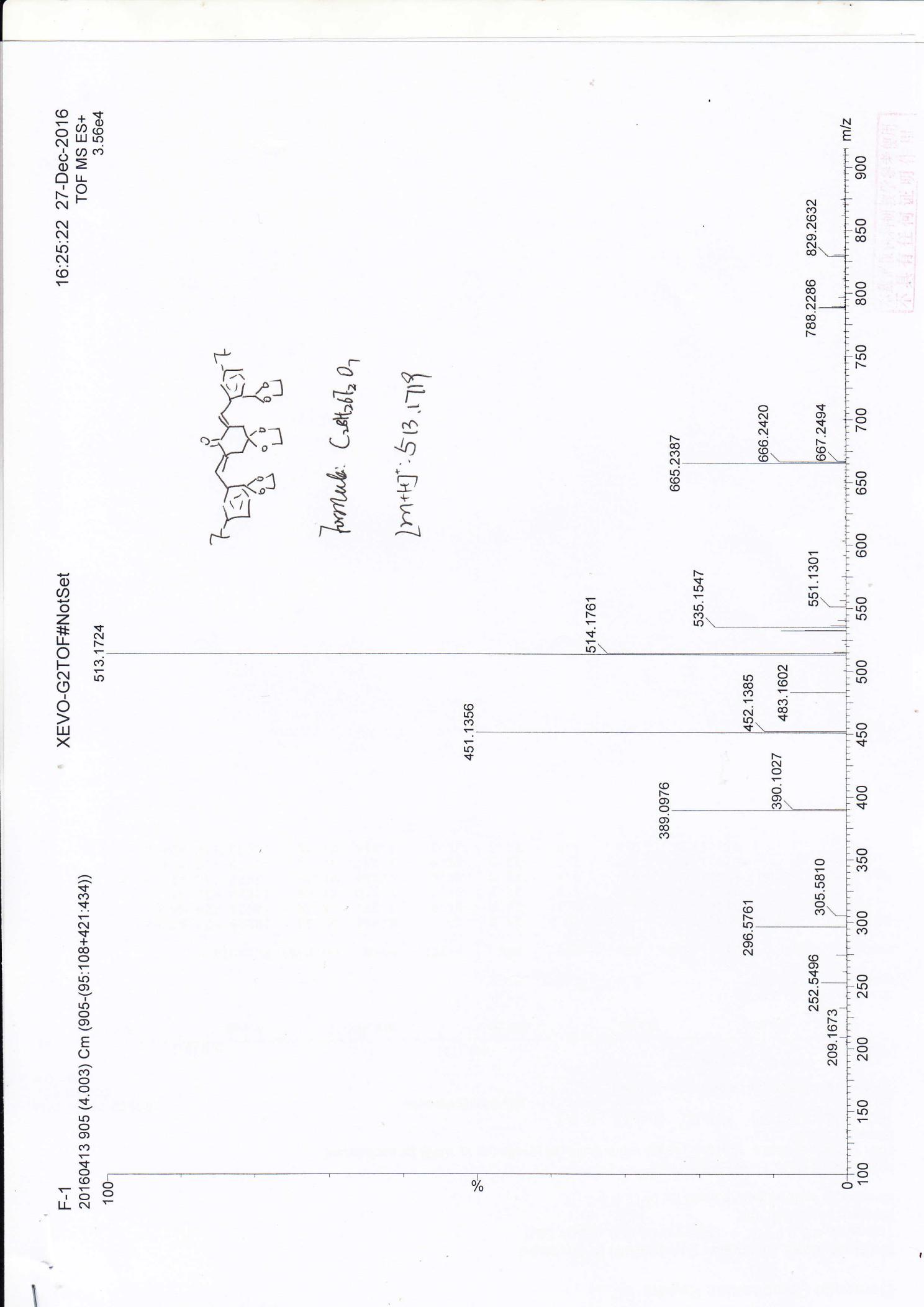
_**

**Fig S12. The HR-MS of compound 4.**


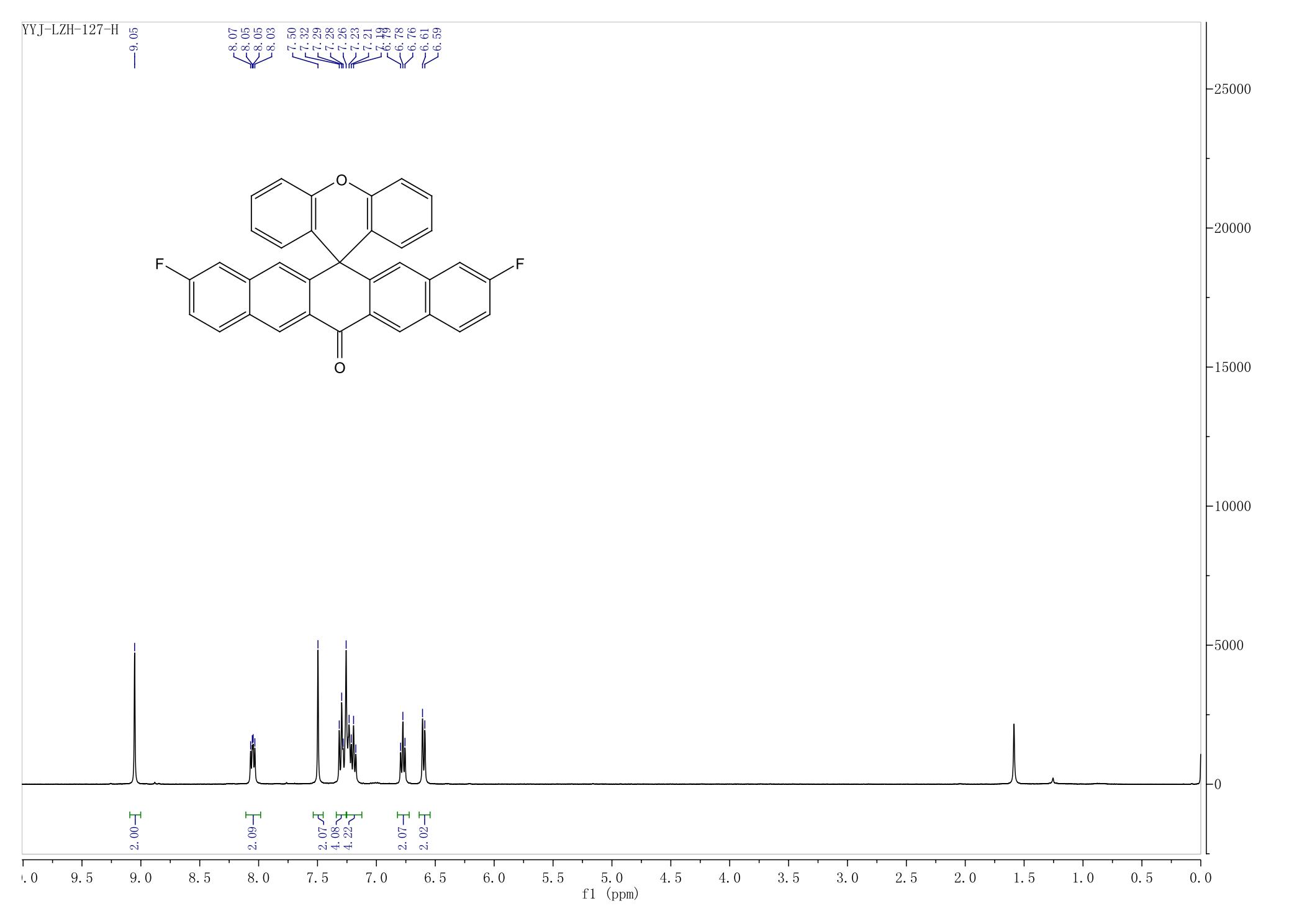


**Fig S13. The ^1^H-NMR of compound 5 in CDCl_3_.**

**
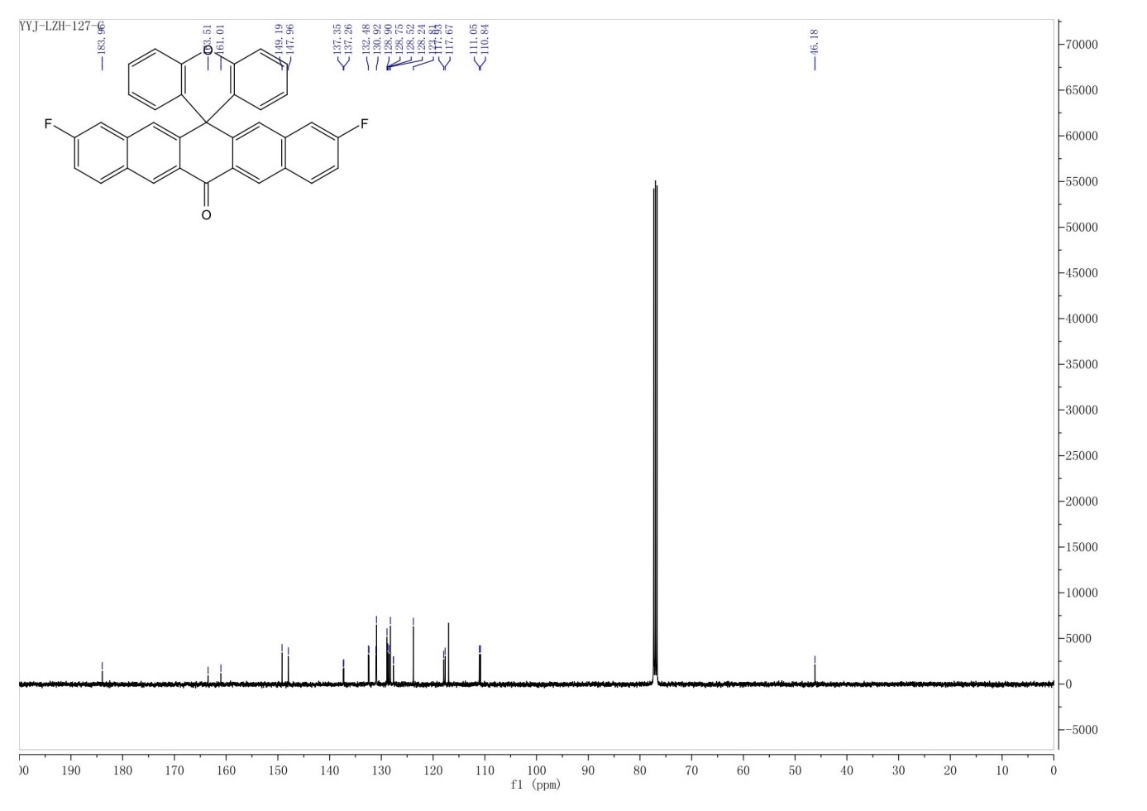
**

**Fig S14. The ^13^C-NMR of compound 5 in CDCl_3._**

**
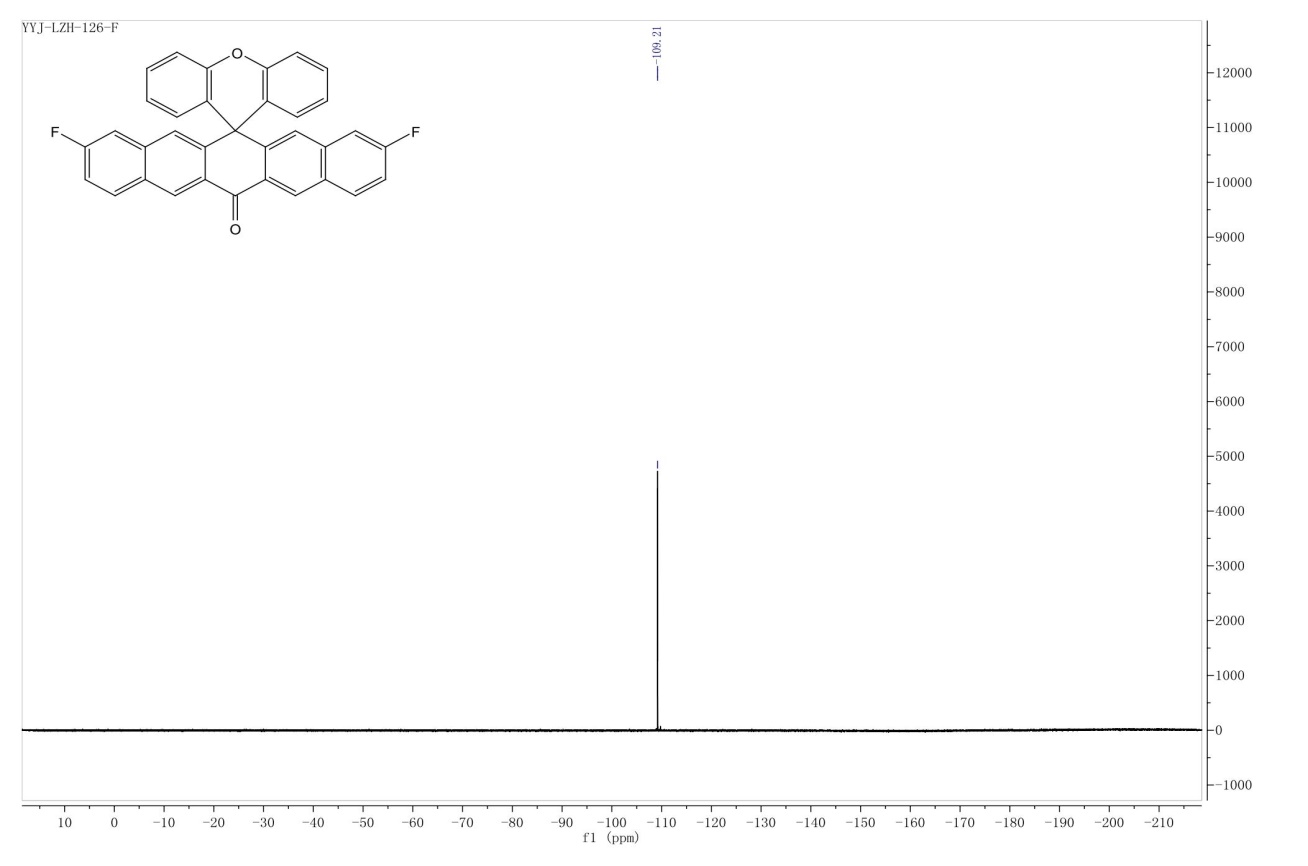
**

**Fig S15. The ^19^F-NMR of compound 5 in CDCl_3._**

**_
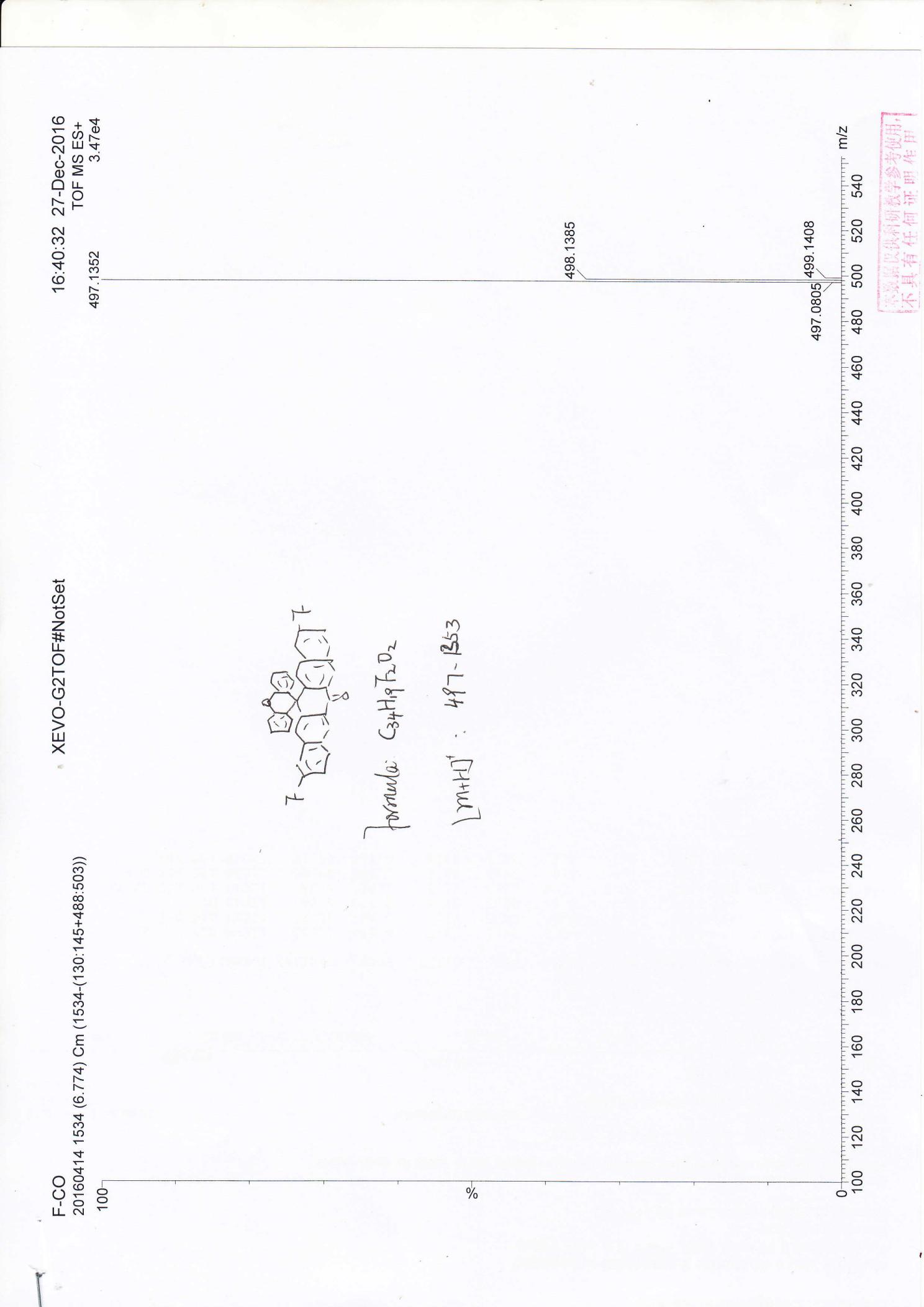
_**

**Fig S16. The HR-MS of compound 5.**

**
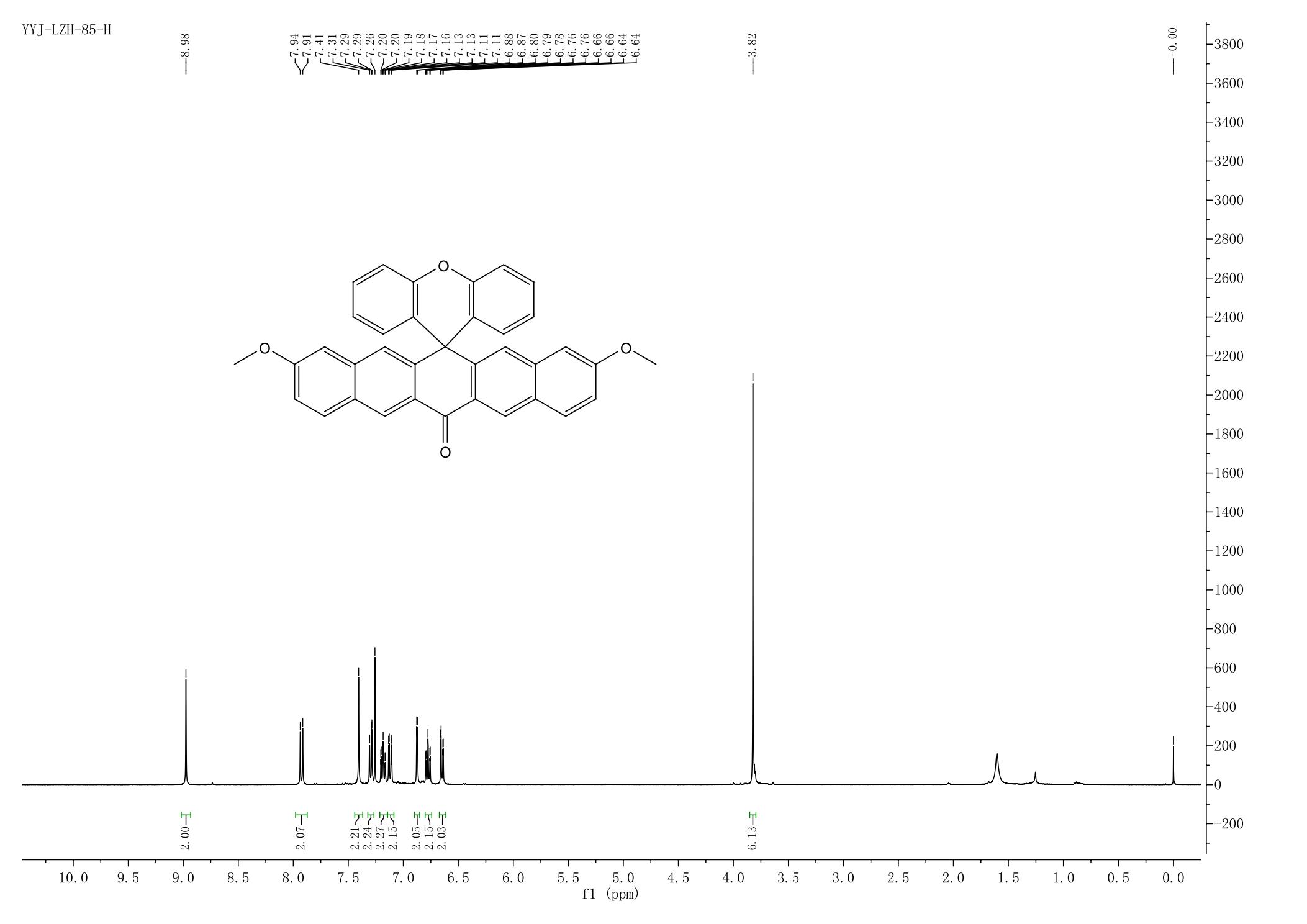
**

**Fig S17. The ^1^H-NMR of compound 6 in CDCl_3_.**

**
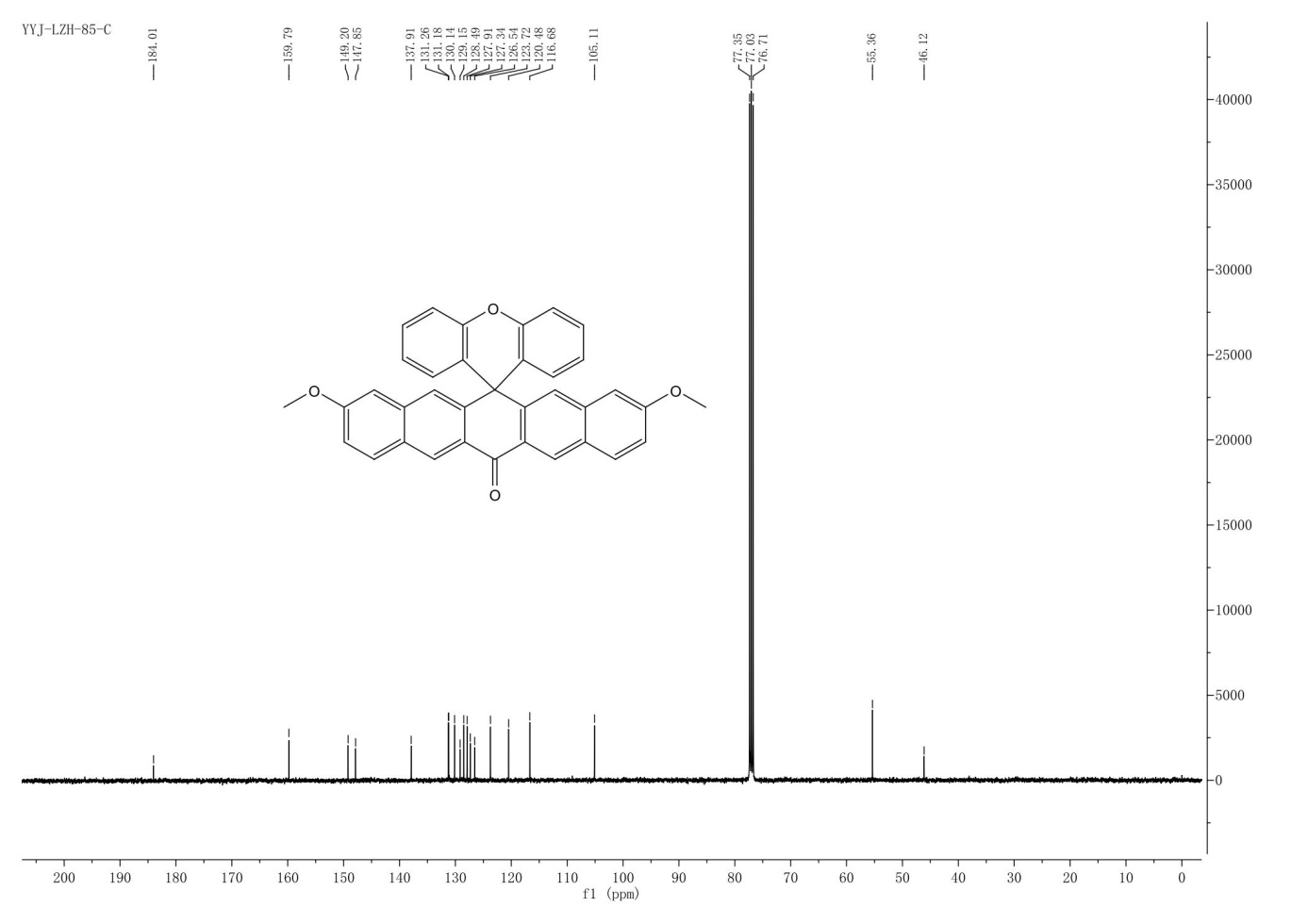
**

**Fig S18. The ^13^C-NMR of compound 6 in CDCl_3._**

**
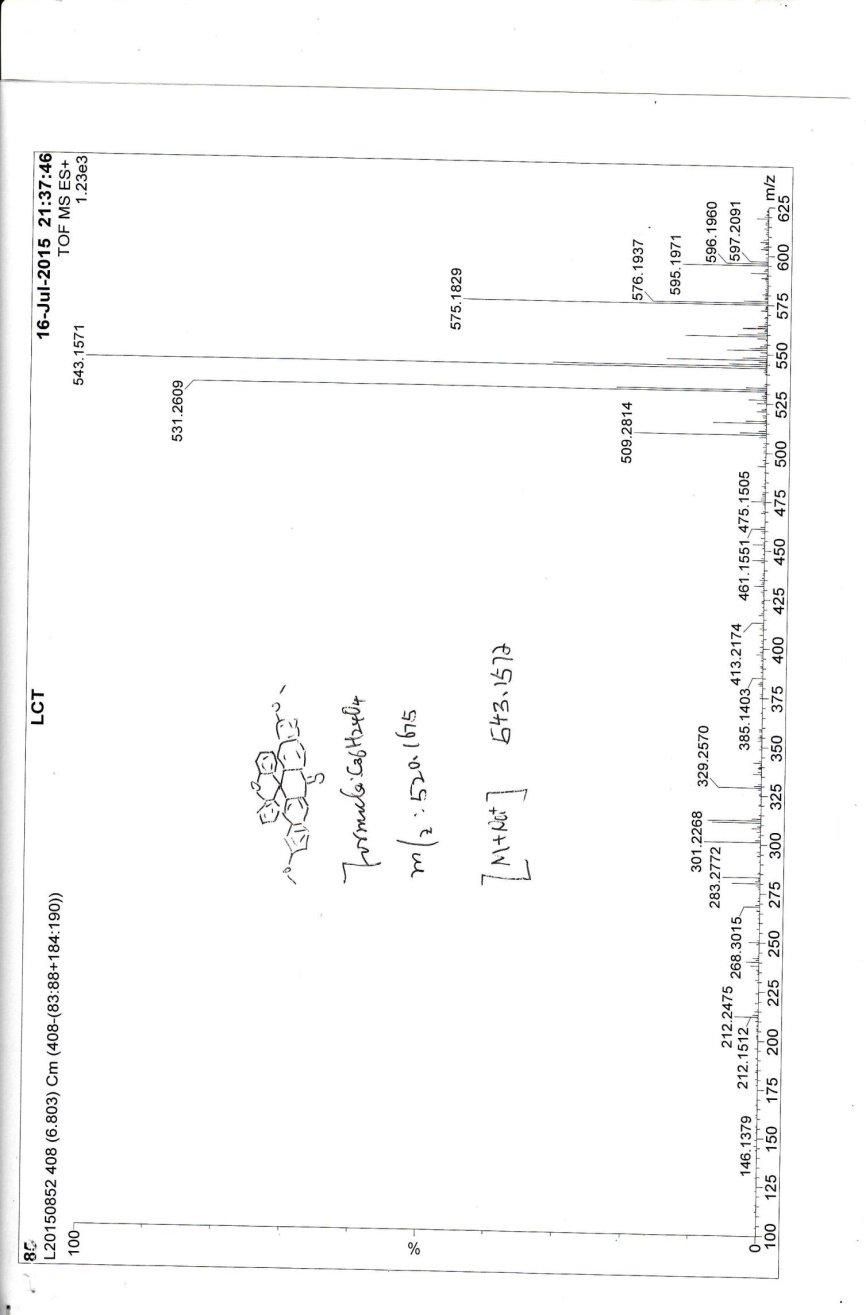
**

**Fig S19. The HR-MS of compound 6.**

**
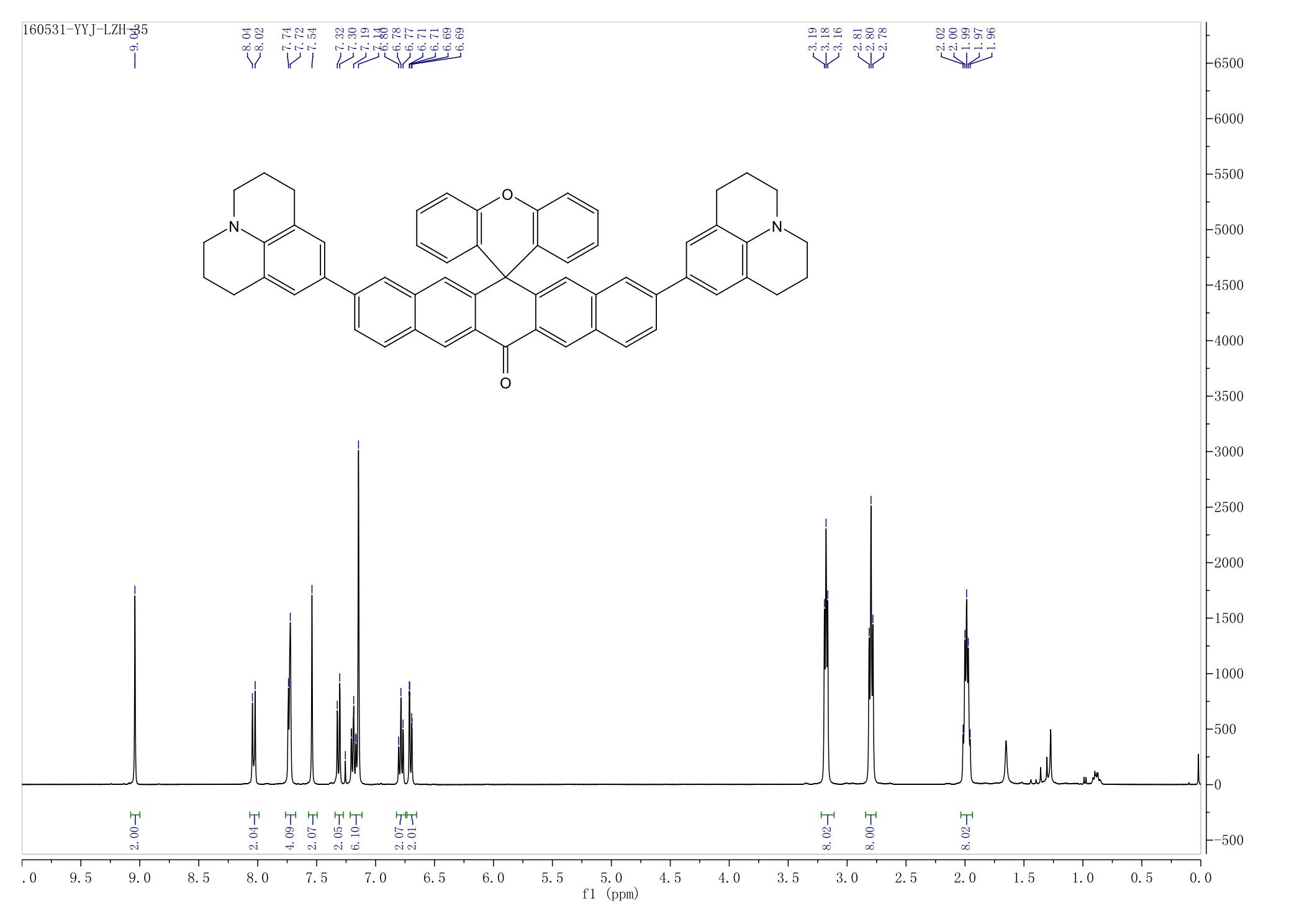
**

**Fig S20. The ^1^H-NMR of compound VPZ2 in CDCl_3_.**

**
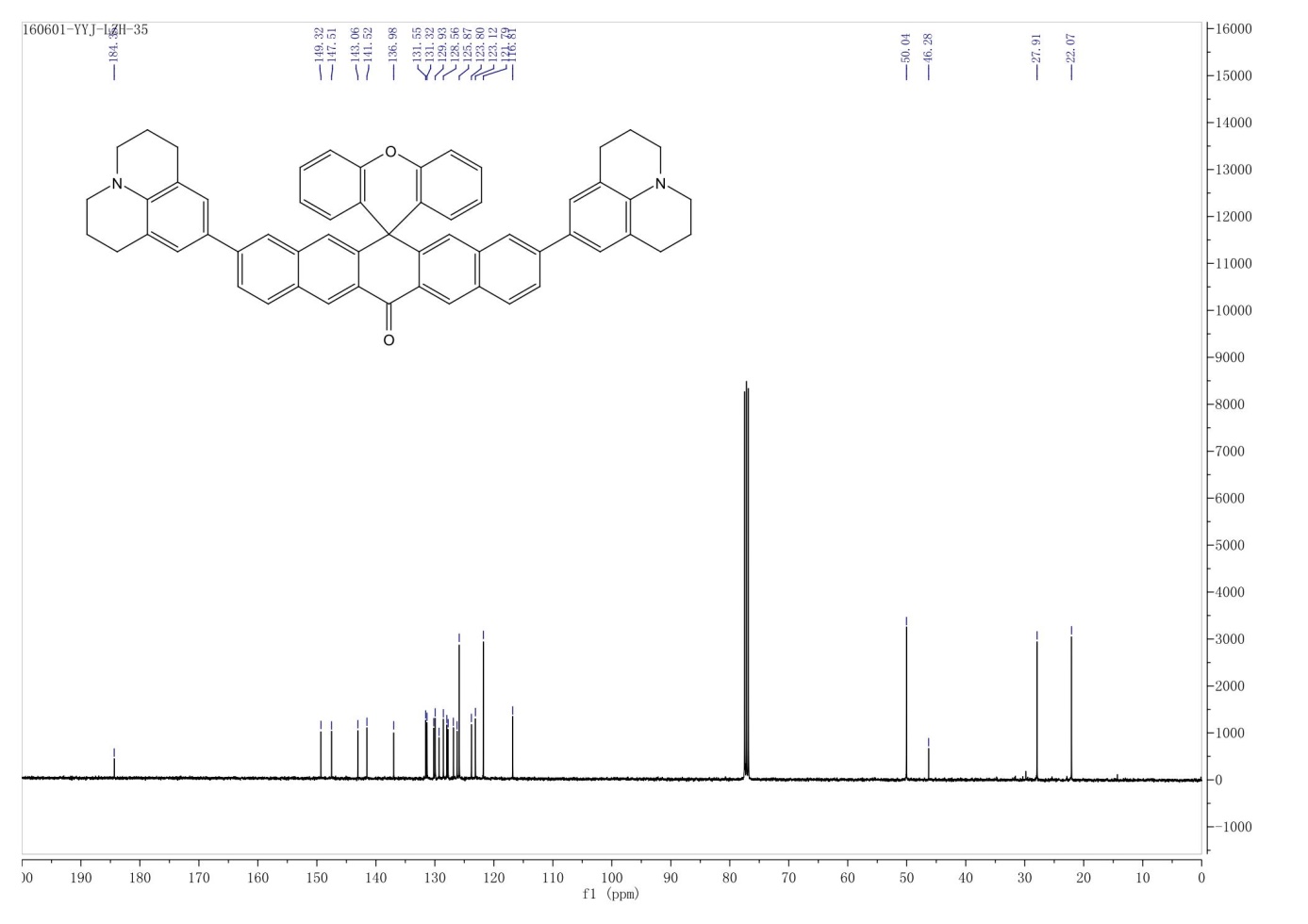
**

**Fig S21. The ^13^C-NMR of compound VPZ2 in CDCl_3._**


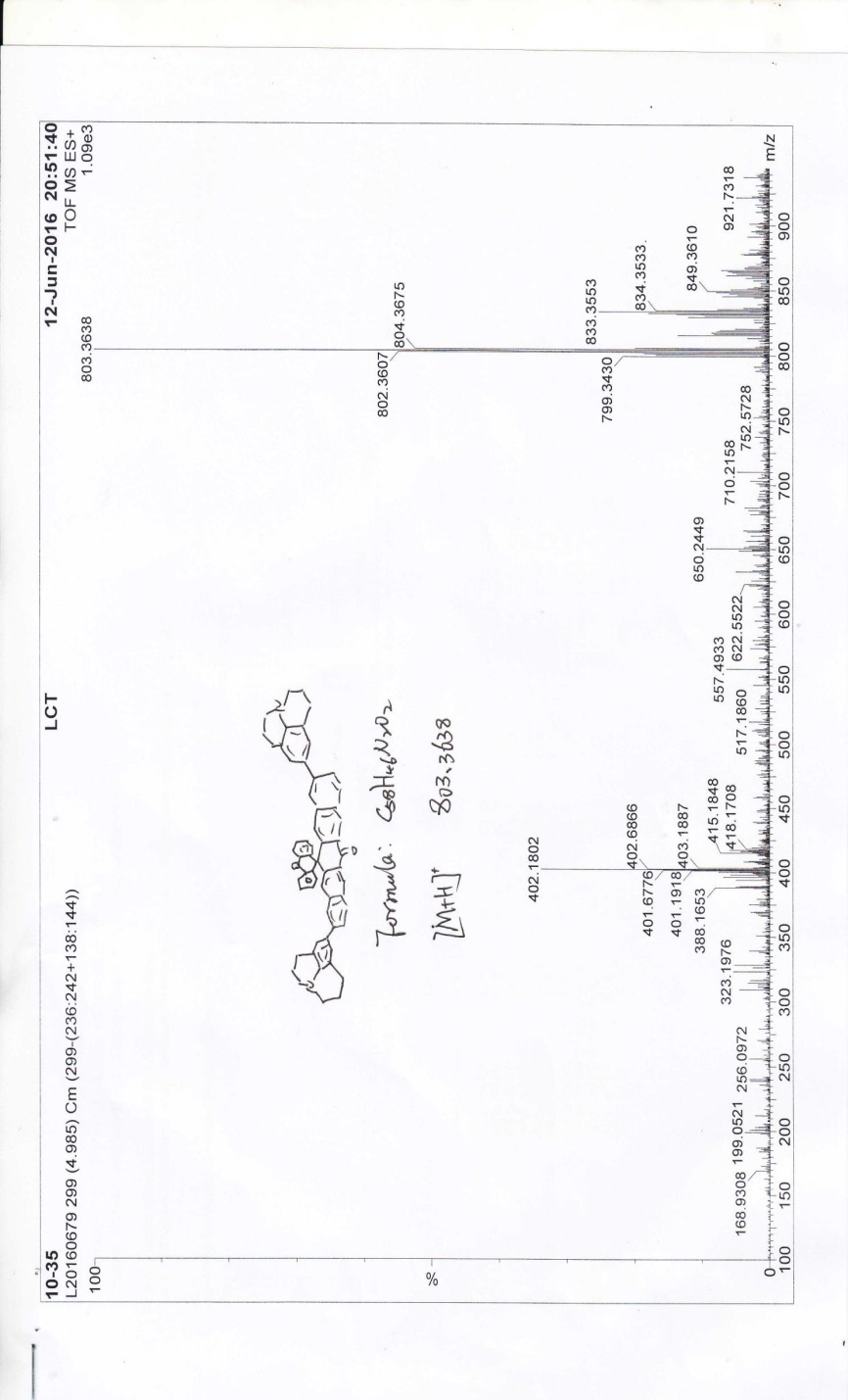


**Fig S22. The HR-MS of compound VPZ2.**

**
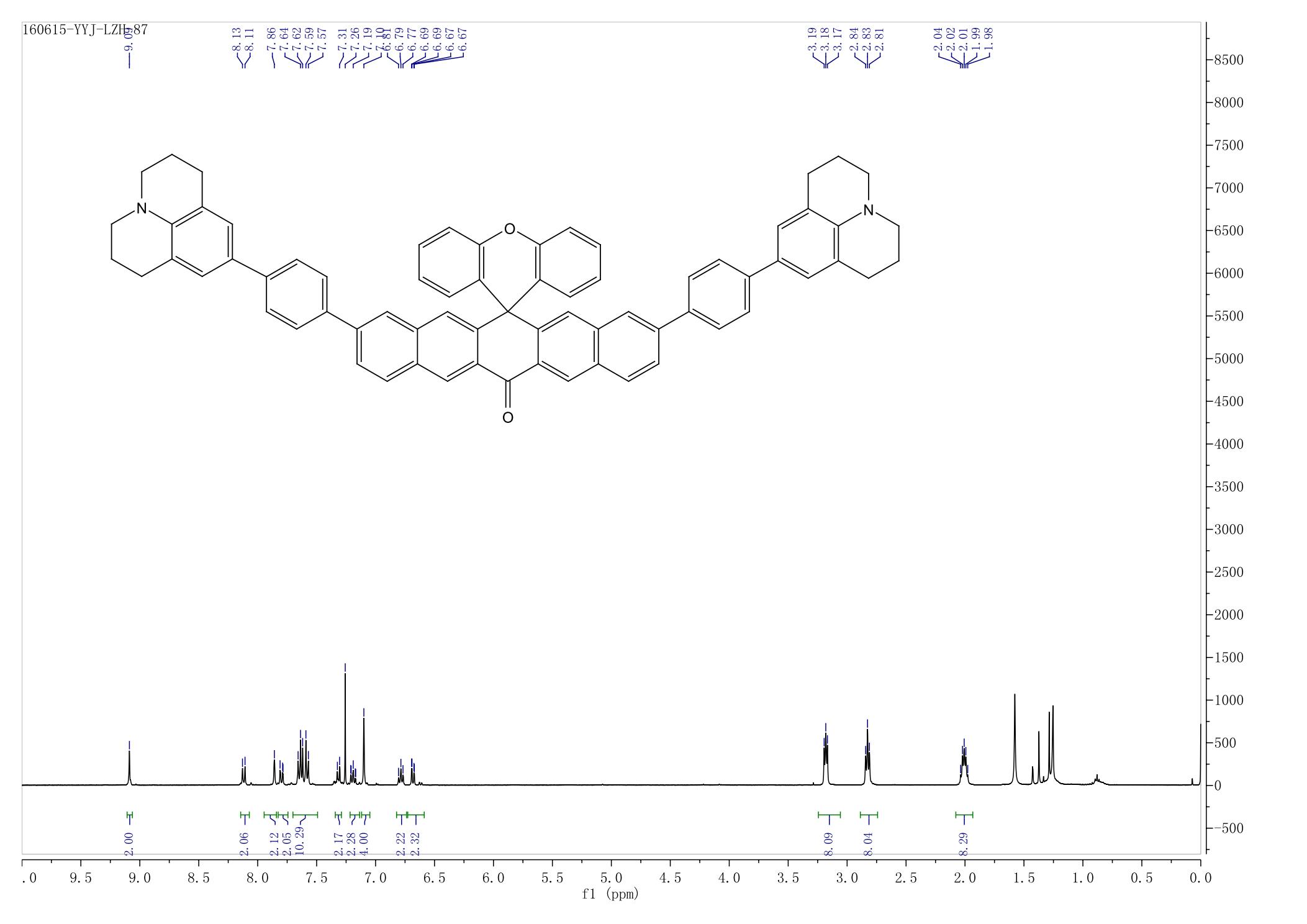
**

**Fig S23. The ^1^H-NMR of VPZ3 in CDCl_3_.**

**
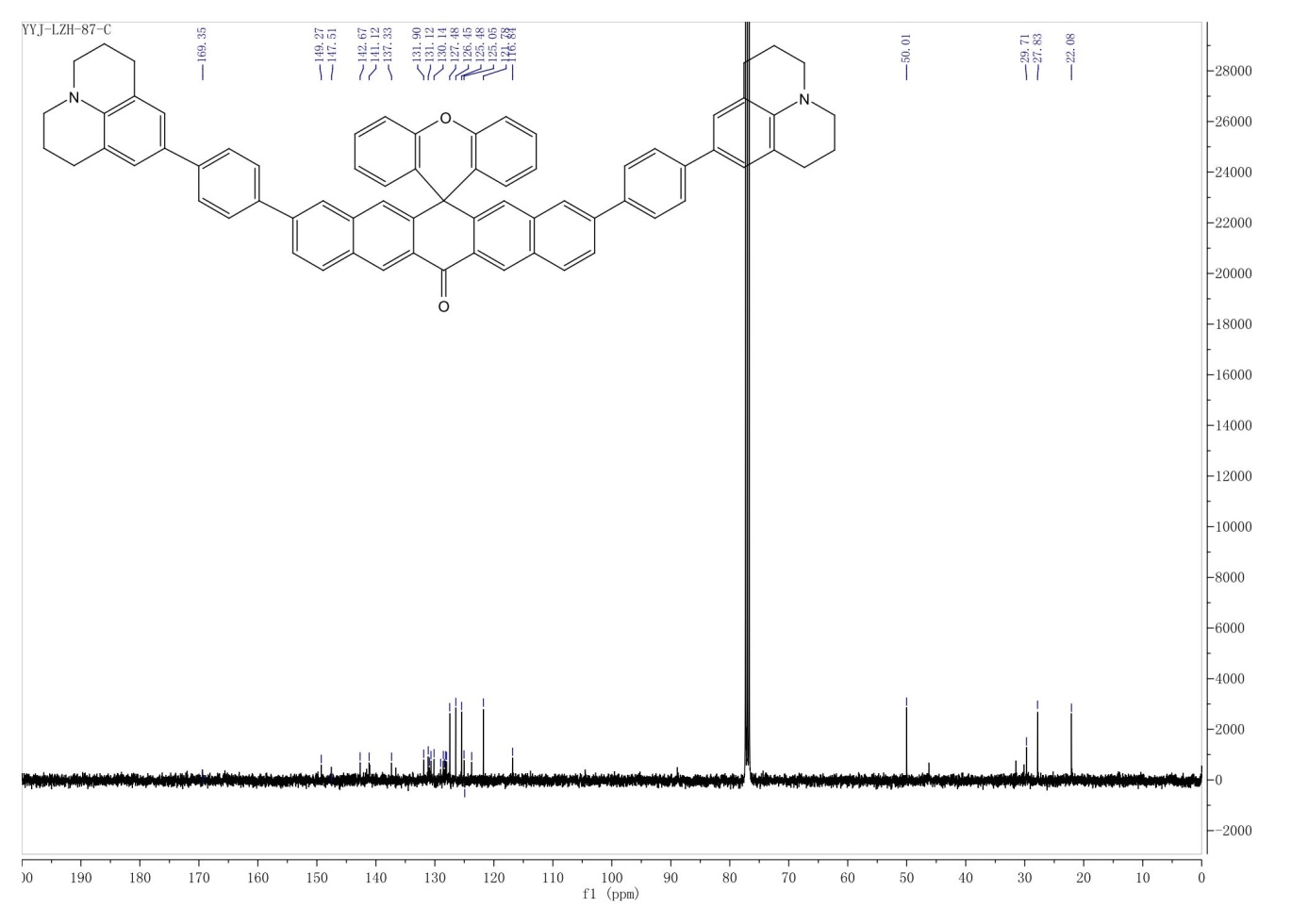
**

**Fig S24. The ^13^C-NMR of VPZ3 in CDCl_3._**


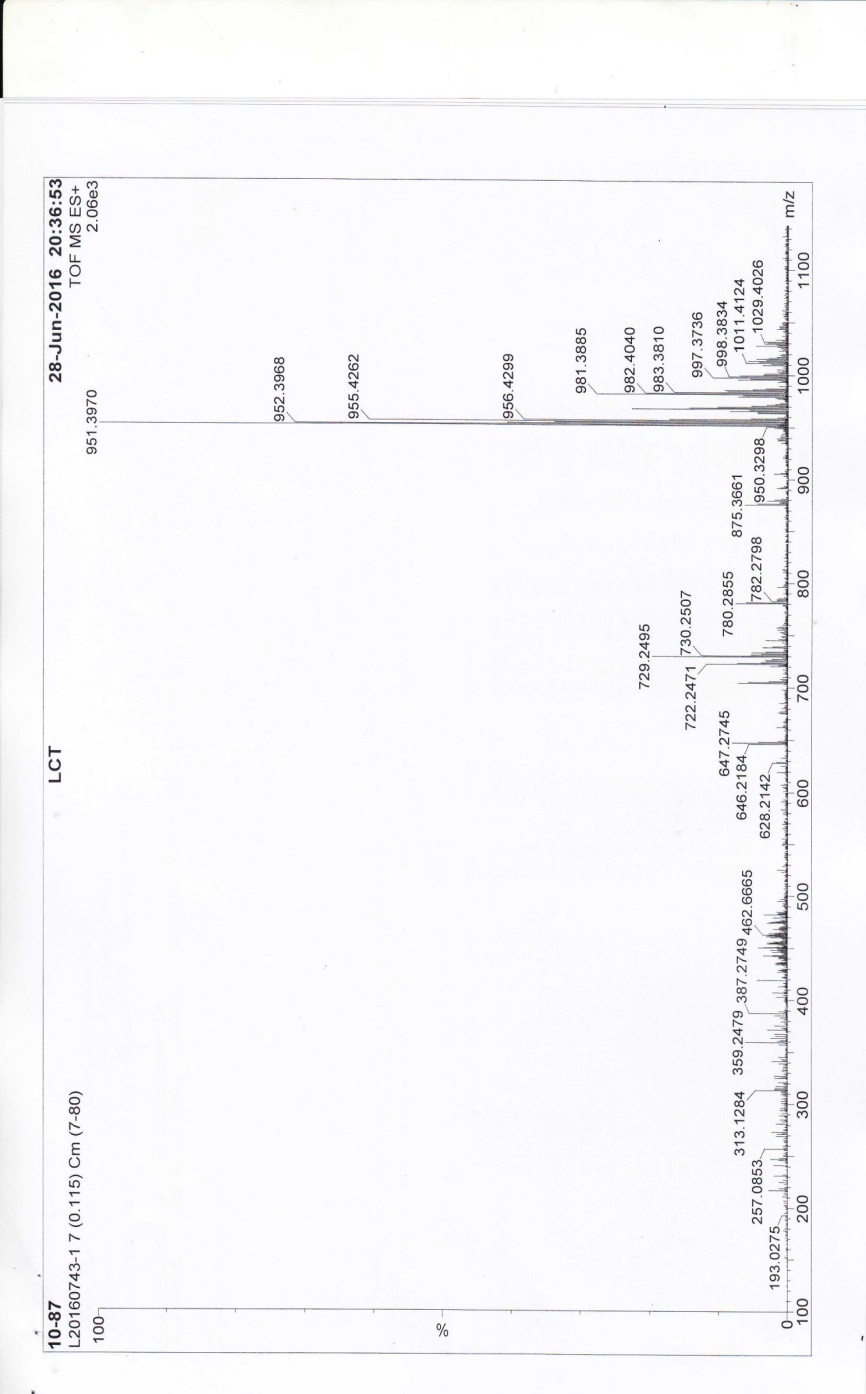


**Fig S25. The HR-MS of VPZ3.**

Identification code mo_dm15781_0m

Empirical formula C34.50 H19 Cl F2 O2

Formula weight 538.95

Temperature 296.15 K

Wavelength 0.71073 Å

Crystal system Monoclinic

Space group P 1 21/n 1

Unit cell dimensions a = 11.558(3) Å a= 90°.

b = 16.573(5) Å b= 109.866(5)°.

c = 13.868(4) Å g = 90°.

Volume 2498.4(12) Å3

Z 4

Density (calculated) 1.433 Mg/m3

Absorption coefficient 0.201 mm-1

F(000) 1108

Crystal size 0.3 x 0.25 x 0.2 mm3

Theta range for data collection 1.987 to 26.999°.

Index ranges -16<=h<=16, -23<=k<=20, -19<=l<=17

Reflections collected 25347

Independent reflections 5459 [R(int) = 0.0531]

Completeness to theta = 26.000° 100.0 %

Absorption correction Semi-empirical from equivalents

Max. and min. transmission 0.7461 and 0.6586

Refinement method Full-matrix least-squares on F2

Data / restraints / parameters 5459 / 0 / 371

Goodness-of-fit on F2 1.040

Final R indices [I>2sigma(I)] R1 = 0.0519, wR2 = 0.1358

R indices (all data) R1 = 0.1182, wR2 = 0.1760

Extinction coefficient 0.0048(9)

Largest diff. peak and hole 0.307 and -0.199 e.Å-3

**Fig S26. The X-ray single crystal of 5.**

Figure S27 fluorescence lifetime of **VPZ3** in different viscosity solutions.


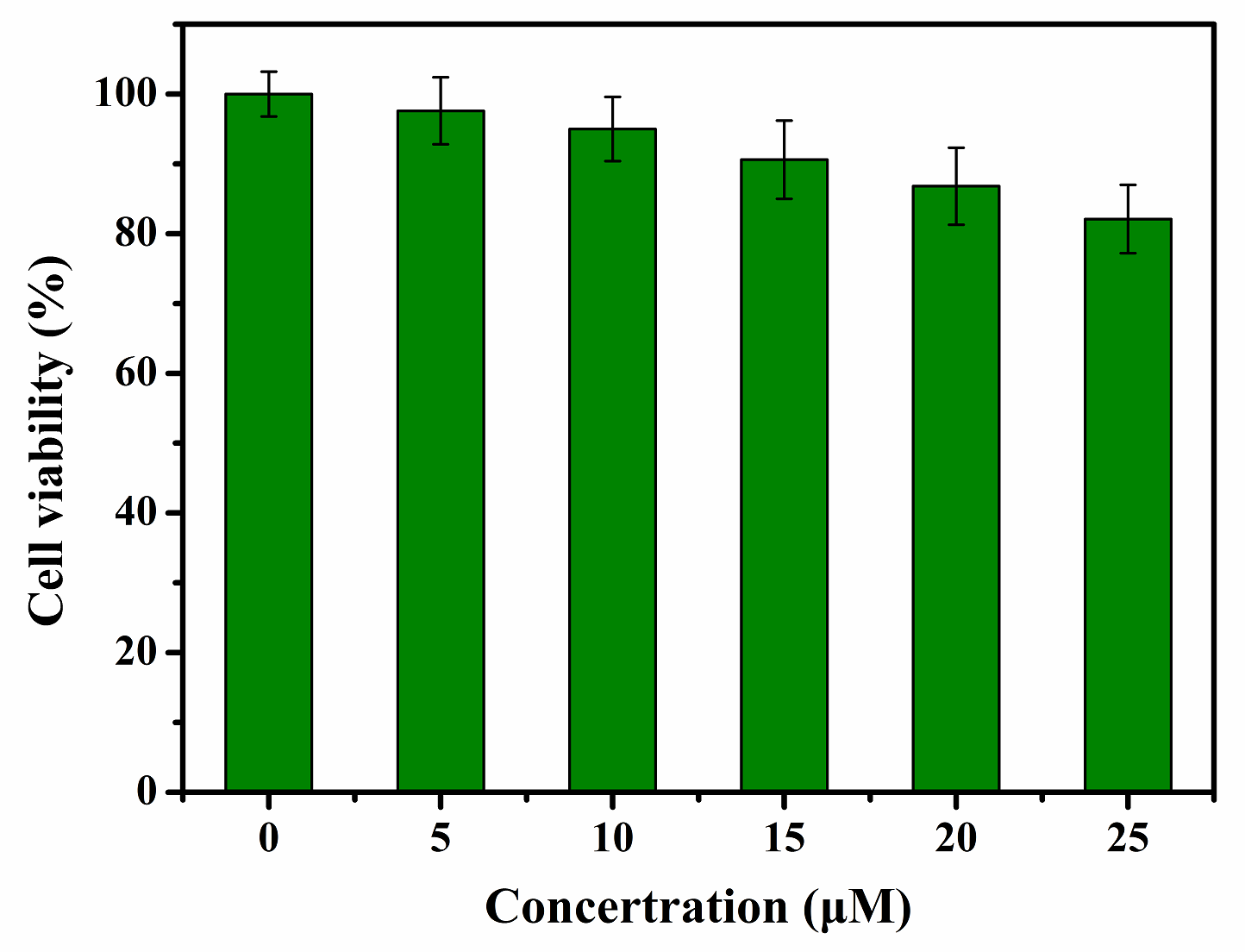


**Figure S28.** Cytotoxicity data of **VPZ3** (HepG2 cells incubated for 24 h).


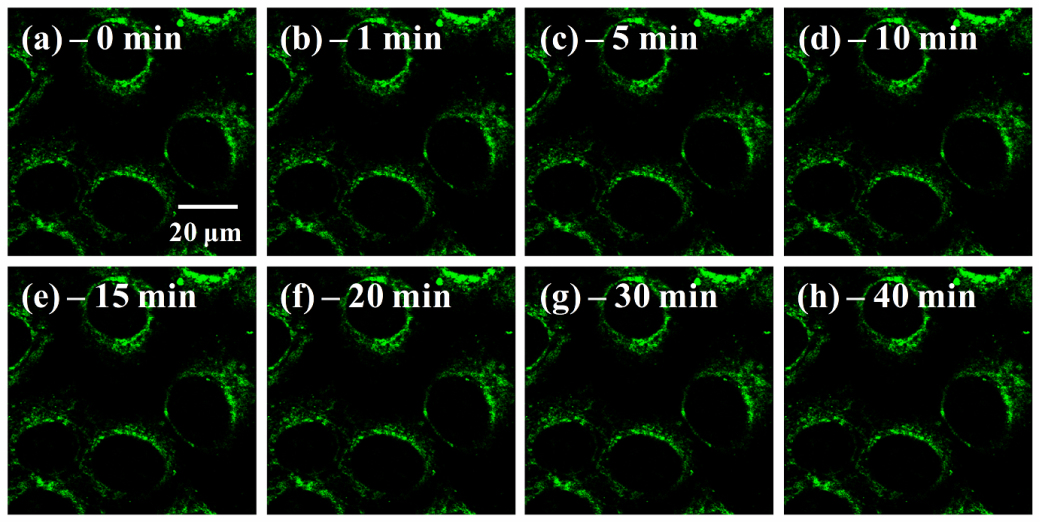


**Figure S29** (a-h) Two-photon confocal images of HepG2 cells incubated with 10 μM **the probe** at different time points, *λ*_ex_ = 770 nm, emission wavelength from 500 nm to 540 nm. Scale bars: 20 μm.
